# The High Impact of *Staphylococcus aureus* Biofilm Culture Medium on In Vitro Outcomes of Antimicrobial Activity of Wound Antiseptics and Antibiotic

**DOI:** 10.3390/pathogens10111385

**Published:** 2021-10-26

**Authors:** Justyna Paleczny, Adam Junka, Malwina Brożyna, Karolina Dydak, Monika Oleksy-Wawrzyniak, Daria Ciecholewska-Juśko, Ewelina Dziedzic, Marzenna Bartoszewicz

**Affiliations:** 1 Department of Pharmaceutical Microbiology and Parasitology, Faculty of Pharmacy, Wroclaw Medical University, 50-556 Wroclaw, Poland; justyna.paleczny@student.umed.wroc.pl (J.P.); malwina.brozyna@gmail.com (M.B.); karolina.dydak@umed.wroc.pl (K.D.); monika.oleksy@umed.wroc.pl (M.O.-W.); m.bartoszewicz@op.pl (M.B.); 2 Department of Microbiology and Biotechnology, Faculty of Biotechnology and Animal Husbandry, West Pomeranian University of Technology, 70-311 Szczecin, Poland; daria.ciecholewska@zut.edu.pl; 3 Faculty of Medicine, Lazarski University, 02-662 Warszawa, Poland; ewelinadziedzic82@gmail.com

**Keywords:** biofilm, *Staphylococcus aureus*, wound infection, exudate, culture media, antiseptics

## Abstract

The staphylococcal biofilm-based infections of wounds still pose a significant therapeutical challenge. Treated improperly, they increase the risk of limb amputation or even death of the patient. The present algorithms of infected wound treatment include, among others, the application of antiseptic substances. In vitro wound biofilm models are applied in order to scrutinize their activity. In the present work, using a spectrum of techniques, we showed how the change of a single variable (medium composition) in the standard in vitro model translates not only to shift in staphylococcal biofilm features but also to the change of efficacy of clinically applied wound antimicrobials such as octenidine, polyhexamethylene biguanide, chlorhexidine, hypochlorite solutions, and locally applied gentamycin. The data presented in this study may be of a pivotal nature, taking into consideration the fact that results of in vitro analyses are frequently used to propagate application of specific antimicrobials in hospitals and ambulatory care units.

## 1. Introduction

Chronic wound infection has a significant impact not only on a patient’s health, but also on their social and economic status. Untreated or treated improperly, infected chronic wound is a factor significantly increasing risk of limb amputation or development of systemic, life-threatening inflammatory symptoms. The treatment of chronic wound infection is still considered a challenge for contemporary medicine and includes application of time-consuming algorithms involving actions performed by multidisciplinary medical team [1]. 

Among the numerous species of pathogens causing wound infections, *Staphylococcus aureus* is one of the most commonly isolated. This Gram-positive coccus is considered to be the one of the leading etiological factors of wound infections [2]. This opportunistic and ubiquitous pathogen displays a high virulence potential that allows it to avoid immune system answer and, to a major extent, antibiotic therapy [3]. One of the specific virulence factors enabling *S. aureus* adaptation to the environment of chronic wound is its ability to form biofilm [4]. This multi-cellular society of aggregated microorganisms is enclosed within self-produced extracellular matrix (ECM). The matrix accounts for even 90% of biofilm’s dry mass and it may be composed of proteins, glycoproteins, polysaccharides, and extracellular DNA [4]. The ECM displays protective and nutritional functions; moreover, it allows bacterial cells to form communities of high spatial density and to exchange virulence factors, metabolites, or messenger molecules. Importantly, ECM composition depends on genetic and environmental factors [5]. The chronic wounds, due to the presence of necrotic tissues and disturbed efficacy of immune system, are considered a highly favorable environment for biofilm development [6]. 

The grown, mature biofilm consists of metabolically differentiated subpopulations displaying various need for oxygen, nutrients, and differentiated level of tolerance towards antibacterial agents [4]. During the process of its development or in result of mechanical disruption, biofilm may disperse and colonize neighboring areas in order to establish new biofilms [5]. It was revealed that bioactive compounds produced by *S. aureus* biofilm impair migration and proliferation of keratinocytes in chronic wounds [7]. It is therefore widely accepted that biofilm presence may hinder or even deteriorate the process of wound healing [6,8]. Therefore, it is of paramount importance to fully recognize and understand the role of biofilm in wound infection in order to develop appropriate counter-measures [9,10]. Presently, they consist mostly of debridement, active dressings, and antimicrobials of various kinds. The latter ones can be divided into antibiotics (applied as systemic prophylaxis, with exception of locally administered gentamycin) and topically used antiseptics. 

It was proven in laboratory in vitro studies that biofilms demonstrate even 1000× higher tolerance to various antimicrobial agents than planktonic (non-adhered) cells [5] of the same microbial strain. The recent reports also indicate that biofilm (understood as structure consisting of cells and ECM) is strongly affected by the environment [11]. Bearing in mind the fact of protective function displayed by ECM, we may assume that differences in its composition (being result of impact of different environment) may translate into different levels of efficacy displayed by wound antimicrobials against it. 

The high conditions imposed to wound antimicrobials are result of high biofilm tolerance and persistence. The most important properties of wound antimicrobial include, among others, a wide spectrum of antimicrobial and antibiofilm activity, no risk of induction of microbial resistance, ability to improve wound healing, or lack/low cyto- and genotoxicity [12]. 

Polyhexamethylene biguanide hydrochloride (polyhexanide, PHMB) is one such commonly applied wound and skin lesion antiseptic. PHMB binds to lipopolysaccharides and peptidoglycan, disrupting the bacterial cell membrane [13]. Recently, Chider et al. proved that PHMB is able also to penetrate through microbial cell wall and binding to chromosomes, resulting in DNA function impairment [14]. The antimicrobial spectrum of PHMB involves Gram-positive and Gram-negative bacteria, including multi-drug-resistant and spore-forming strains (PHMB activity against spores is still a matter of scientific debate [15]). According to some research teams, even a low concentration of PHMB eradicates biofilm efficiently [16]. 

Octenidine dihydrochloride (OCT) is also a widely used wound antiseptic. This molecule’s binding to the negatively charged elements of the cell wall triggers cell wall disruption and bacterium lysis [17]. OCT is considered highly effective against biofilm formed by Gram-positive or Gram-negative strains. Similarly to PHMB, the OCT spectrum of activity does not include spore forms [17,18]. 

In turn, the mechanism of action of chlorhexidine gluconate (CHX) depends on its concentration. Applied as an antiseptic, CHX inhibits enzymes and causes precipitation of cytoplasmic elements [19]. CHX has a broad spectrum of activity and effectively eradicates biofilm even in low concentrations. Nevertheless, more and more cases of CHX-resistant species are notified [20]. 

Iodine povidone (PVP-I) has a different mechanism of action from the above-mentioned substances. PVP-I transports a molecule of iodine into the cell, where it binds with proteins, nucleotides, amino acids, and fatty acids and leads to cell death. PVP-I is effective against vegetative and spore forms of bacteria and fungi [21]. Moreover, high ability of this antiseptic to eradicate biofilm has been confirmed [22,23]. 

Combinations of sodium hypochlorite (NaOCl) and hypochlorous acid (HOCl) are new formulations of antiseptic agents used for centuries. Antimicrobial activity relies on increasing the permeability of cells’ walls, causing water to inflow into the cells that results in cell lysis [24]. The efficacy of hypochlorites is a controversial subject. There are scientific reports indicating both strong as well as poor antimicrobial/antibiofilm properties of these antiseptics [25,26]. Recent data, provided by Severing et al. [27] and Krasowski et al. [18], suggest that low concentration (80 parts per milion) of NaOCl/HOCl molecules applied for wound treatment may act instead through flushing microbes out of the wound and not by direct bactericidal effect. 

The systemic antibiotics are not recommended in wound treatment because of the issues related with increasing microbial resistance. One of the few antibiotics allowed to be locally applied to wounds is gentamicin sulfate-containing cream or sponge. Gentamycin sulfate displays a bactericidal activity (against Gram-positive and Gram-negative bacteria). It binds to ribosomal subunit inhibiting protein biosynthesis [28]. Maczynska et al. indicated that high concentrations of gentamycin applied in sponge act effectively against biofilm formed by various opportunistic pathogens [29].

In in vitro analyses of antiseptics’ activity, which are a necessary step, preceding studies on animal model and subsequently on humans, the microbial biofilm is frequently cultured in one type of standard microbiological medium (in specific cases supplemented by protein/blood content). It should be noticed that regardless of whether it is Muller–Hinton broth (indicated by EUCAST for antibiotic activity assessment), brain heart infusion, or tryptic soy broth, the molecular compositions of these liquid media do not reflect the composition of wound fluid [30,31,32], in which wound biofilm actually develops. Therefore, the questions aimed in this study were as follows: (i) do applications of various media translate into different compositions of microbial biofilm of the same strain? and (ii) do biofilms of the same strain, grown in various media, display various tolerances to the same type of applied antimicrobials? Being aware of the fact that results of in vitro analyses are frequently used to propagate application of specific antimicrobials in hospitals and ambulatory care units, we found these above-mentioned questions to be of a pivotal nature.

To answer them, we tested the ability of *Staphylococcus aureus* strains, isolated from chronic wounds, to form biofilm in three different media: tryptic soy broth, tryptic soy broth supplemented with 1% glucose, and Dulbecco′s modified Eagle′s medium with 10% fetal bovine serum, and subsequently assessed the correlation between medium applied and antimicrobial/antibiofilm efficacy of commonly used antimicrobial agents: polyhexanide, octenidine, iodine povidone, hypochlorite/hypochlorous acid, gentamycin.

## 2. Results

**Figure 1:**
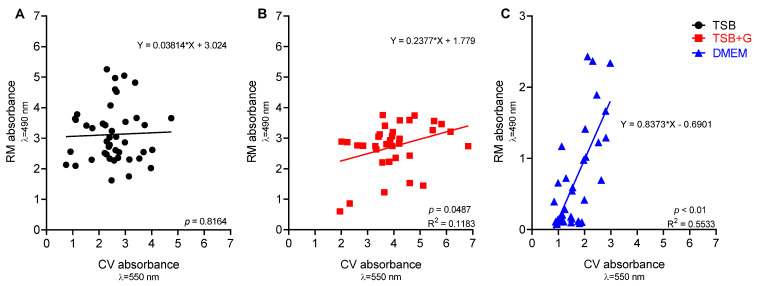
Scatter plots of correlations of ability to form biofilm (CV absorbance) and metabolic activity (RM absorbance) for strains cultivated in three media: (**A**) tryptic soy broth TSB, (**B**) tryptic soy broth supplemented with 1% glucose TSB+G, and (**C**) Dulbecco’s modified Eagle’s medium (DMEM) high glucose; R^2^—the coefficient of determination, *p*—probability level.

In the first experimental line, the ability of staphylococcal strains to form biofilm was verified and visualized by means of epifluorescent/confocal microscopy (Figures S1–S3). Next, the biomass and cells’ metabolic activity in the biofilm formed in various culture media were assessed. Results indicated that not only all strains were able to form biofilm in applied in vitro settings, but they also showed significant correlation (*p* < 0.05) between level of biomass and metabolic activity for strains cultured in TSB+G and DMEM, but not in TSB medium (Figure 1). Coefficients of correlations were determined as average for TSB+G (R = 0.34) and high for DMEM cultures (R = 0.66). The determination coefficients indicated low curve fitting for TSB+G (R^2^ = 0.12) and medium for DMEM (R^2^ = 0.55). A linear regression analysis of bacterial viability in biofilm measured by Richard’s method and colony-forming unit (CFU) count was formulated to estimate CFU from the absorbance intensity. A high level of coefficient of determination (R^2^ = 0.71) and a statistical significance (*p* < 0.001) indicate the high suitability of the equation (Figure S4).

**Figure 2:**
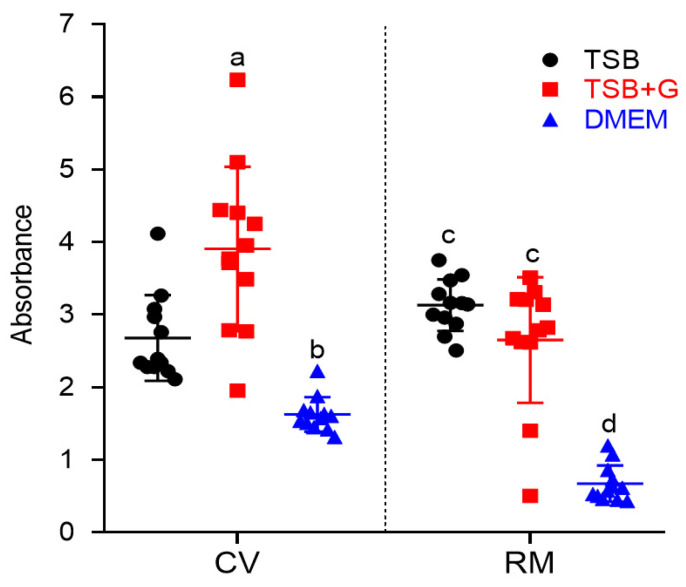
Ability to form biofilm of *Staphylococcus aureus* strains cultivated in three media: tryptic soy broth (TSB), tryptic soy broth with 1% glucose (TSB+G), and Dulbecco’s modified Eagle’s medium (DMEM). Results of two methods: the crystal violet method (CV) and Richard’s method (RM) are presented. An average and standard deviations are marked. Pairs of letters (a/b, c/d) refer to the differences being statistically significant (*p* < 0.05).

**Figure 3:**
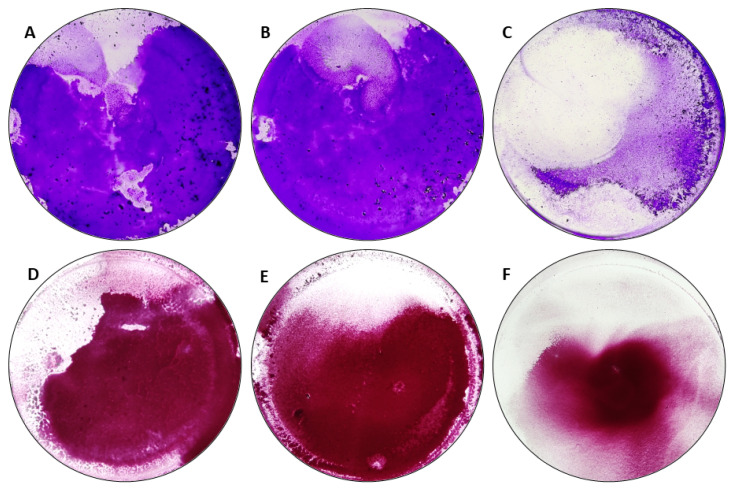
Biofilm formed by *Staphylococcus aureus* ATCC 3538 on a 96-well test plate stained using crystal violet method (CV) (**A**–**C**) and Richard’s method (RM) (**D**–**F**), cultured in tested media: (**A**,**D)** tryptic soy broth (TSB); (**B**,**E**) tryptic soy broth with 1% glucose (TSB+G); (**C**,**F**) Dulbecco’s modified Eagle’s medium (DMEM) high glucose.

The next step aimed to assess, whether the same strain, cultured in different media, formed different levels of biofilm biomass and metabolic activity. It occurred (Figure 2 and Figure 3) that staphylococcal strains cultured in TSB or TSB+G medium formed biofilm at similar levels. The biofilm cultured in DMEM was significantly weaker (with regard to metabolic activity) than biofilm cultured in TSB or TSB+G and also significantly weaker (with regard to biomass level) than biofilm cultured in TSB+G. Noteworthy, results obtained for biofilms cultured in DMEM displayed higher cohesion than results obtained for biofilms cultured in TSB and TSB+G, where standard deviations of outcomes were even fivefold higher than in DMEM. 

**Figure 4:**
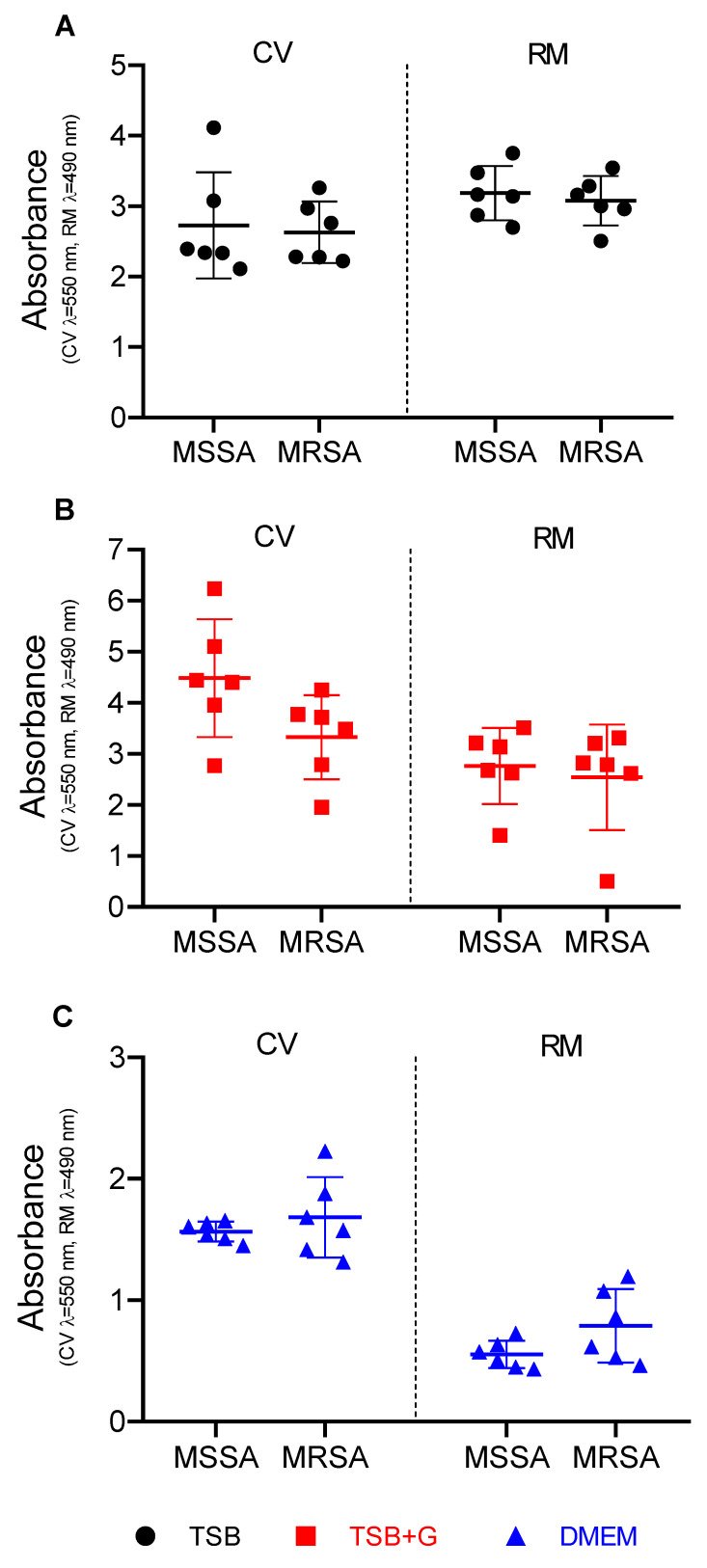
Ability to form biofilm of MSSA—methicillin-susceptible *Staphylococcus aureus* and MRSA—methicillin resistance *Staphylococcus aureus* strains cultivated in three media: (**A**) tryptic soy broth (TSB), (**B**) tryptic soy broth with 1% glucose (TSB+G), and (**C**) Dulbecco’s modified Eagle’s medium (DMEM). Results of two methods: the crystal violet method (CV) and Richard’s method (RM) are presented. An average and standard deviations are marked.

As mentioned in the Materials and Methods Section 4.1, the staphylococci analyzed in this study consisted of an even number of MSSA and MRSA strains. Therefore, in Figure 4 and Figure S5, results presenting ability of biofilm formation in three types of applied media are presented for these two groups of strains displaying various sensitivities to methicillin (and majority of β-lactam antibiotics). The outcome of this analysis revealed that there was no relationship between the ability to form biofilm and resistance to methicillin. 

In the next stage of the experiment, the MIC values of analyzed antimicrobials towards planktonic cells of staphylococcal strains were assessed. All relationships between type of medium, antimicrobial applied, and corresponding MIC value, together with significance levels, are presented in Table 1. 

**Figure 5:**
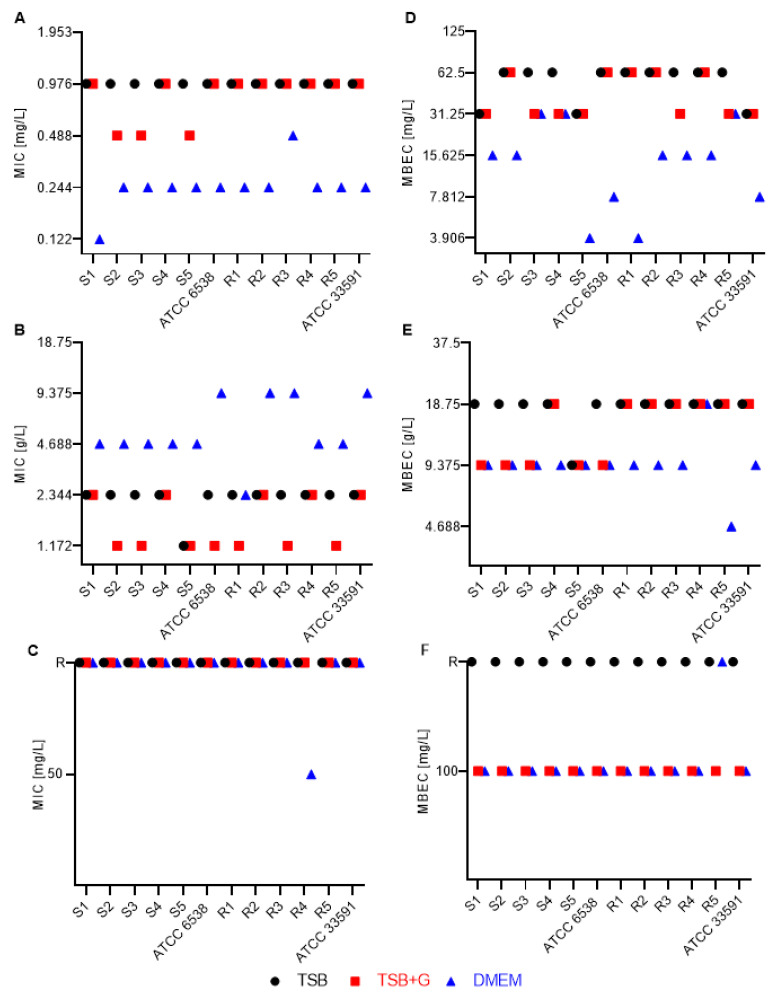
Minimal inhibitory concentration (MIC) (**A**–**C**) and minimal biofilm eradication concentration (MBEC) (**D**–**F**) of (**A**,**D**) octenidine dihydrochloride (OCT); (**B**,**E**) povidone-iodine (PVP-I); (**C**,**F**) 0.01% NaClO/HClO antimicrobial agent (GRAN), of methicillin-susceptible (S1–S5), methicillin-resistant (R1–R2), and American Type Culture Collection (ATCC) *Staphylococcus aureus* strains; ATCC 33591—MRSA strain, ATCC 6538—MSSA strain. Concentrations are presented in mg/L for OCT and GRAN and in g/L for PVP-I; TSB—tryptic soy broth, TSB+G—tryptic soy broth supplemented with 1% glucose, DMEM—Dulbecco’s modified Eagle’s medium. R—resistant in the tested concentration range.

In turn, the particular MIC values recorded for specific staphylococcal strains cultured in specific medium and subjected to activity of antimicrobials are presented in Figure 5 and Figure S6. Results of this analysis indicate that MIC values obtained in TSB+G were the same or varied by just one serial dilution from MICs recorded in the setting where TSB without additional sugar was applied. Contrarily, when DMEM was utilized, the MIC values of OCT, CHX, and PHMB were significantly lower than MICs obtained in the setting where TSB and TSB+G were applied. Of note, the MIC variations in DMEM setting were three (as recorded for OCT and PHMB) or even five (as recorded for CHX) serial dilutions lower than those obtained when TSB was applied (Figure 5 and Figure S6). The opposite relationship was observed for PVP-I, where MICs recorded in DMEM setting were up to three serial dilutions higher than the MICs recorded in TSB setting (Figure 5). Regardless of the medium applied, GRAN and MICR displayed the weakest antimicrobial activity; the significant majority of staphylococcal strains were resistant to these antiseptics in the tested range of concentrations. All tested strains were susceptible to gentamicin (according to the antibiogram results (Table S1)). However, high diversity in MIC values of gentamicin was observed (Figure S6). For most of the tested strains, the MIC of GENTA was 1.953 mg/L in TSB and 3.906 mg/L in TSB+G (Figure S6). The MIC values determined in DMEM did not show any tendency with the values obtained in basic media. The values differed by up to five geometrical dilutions ranging from 0.488 mg/L to even 15.625 mg/L among strains. 

**Figure 6:**
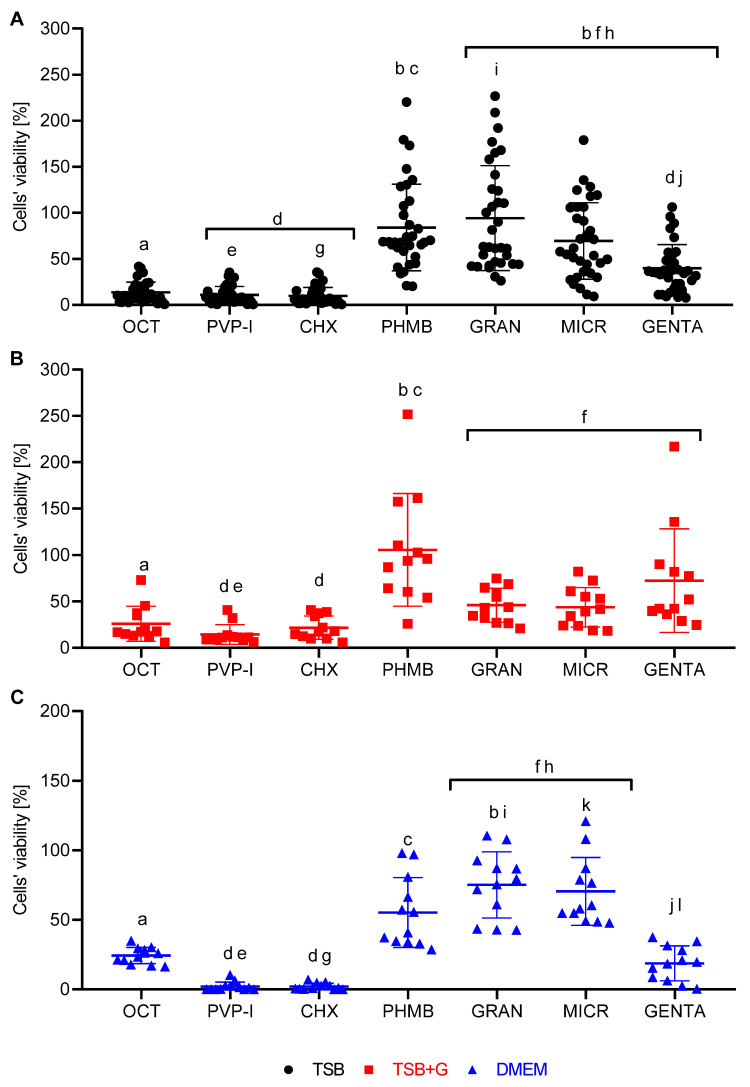
Biofilm eradication activity of octenidine dihydrochloride (OCT), povidone-iodine (PVP-I), chlorhexidine digluconate (CHX), polyhexanide (PHMB), 0.01% NaClO/HClO (GRAN), 0.008% NaClO/HClO (MICR), and gentamycin sulphate (GENTA) in (**A**) TSB, (**B**) TSB+G, and (**C**) DMEM measured with modified antibiofilm dressing’s activity measurement (A.D.A.M.) assay. Pairs of letters a/b, c/d, e/f, g/h, i/j, and k/l refer to significant differences between tested substances. The results are presented as an average of three replications obtained in one experiment, which was repeated two more times.

Next, we assessed the MBEC values of the aforementioned antimicrobials towards biofilm formed in TSB, TSB+G, and DMEM media (Table 2, Figure 6).

MBEC values were higher than MICs (Figure 5) determined in the previous experimental stage. The MBEC values of tested preparations varied depending on the culture medium **(**Table 2, Figure 5). The glucose addition to TSB did not significantly affect the results. The results obtained in TSB and TSB+G were the same or differed in one serial dilution. On the contrary, MBEC values in DMEM were lower by up to five serial dilutions than these recorded in TSB. GRAN and MICR (Figure 5 and Figure S7) showed biofilm eradication activity only in the undiluted preparations in TSB+G and DMEM. All biofilms cultured in TSB showed resistance to 100% concentration of hypochlorite-containing antimicrobials. GENTA concentrations also applied were insufficient for effective eradication of 11/12 staphylococcal biofilms cultured in TSB and TSB+G and 8/12 biofilms cultured in DMEM (Figure S3). To confirm the MBEC values, we assessed a reduction of CFU under the influence of the concentrations range of antimicrobials, on the basis of the linear regression analysis (Figure S4). The MBEC values were established when >99% CFU was reduced comparing to the growth control (Figure S8).

Finally, the biofilm eradication efficacy of tested antimicrobials in each medium was compared using modified antibiofilm dressing’s activity measurement (A.D.A.M.). The test was performed in TSB on all tested strains, and on the basis of the results, one of the most and one of the least resistant strains to each antiseptic were chosen for analysis in TSB+G and DMEM. The final concentrations of OCT and PHMB was 0.57 g/L, CHX was 2.85 g/L, PVP-I was 42.75 g/L, GRAN was 0.057 g/L, MICR was 0.046 g/L, and GENTA was 1.14 g/L. Results are presented as percentage of metabolic activity of cells exposed to antimicrobial compared to the unexposed cells, whose metabolic activity was considered 100% (Figure 6). The highest biofilm eradication activity displayed PVP-I and CHX in all media. However, specific strains treated with PHMB, GRAN, and MICR in TSB, and PHMB and GENTA in TSB+G, were able to grow in the presence of these antimicrobials. This phenomenon manifested in the form of high standard deviations of outcomes obtained. The efficiency of OCT was similar, regardless the medium applied, contrary to PHMB and GENTA, where the eradication activity varied depending on the medium. Both GRAN and MICR in TSB+G and DMEM affected biofilm similarly, despite different concentrations of NaOCl/HOCl used in these two antimicrobials. Of note, the metabolic activity of specific strains after treatment with PHMB, GENTA, GRAN, and MICR in TSB medium was significantly higher than metabolic activity of un-exposed cells; a similar phenomenon was observed in TSB+G medium but only for PHMB and GENTA. In turn, no such trend was in essence observed in cases where DMEM medium was applied (Figure 6).

## 3. Discussion

The pathogens isolated from wound infections display a high ability to form biofilm. Biofilm is a highly organized, cross-linked microbial community, in which, besides metabolically active cells, also slow-growing and dormant cellular phenotypes occur. This cellular differentiation, together with spatial complexity of biofilm, is responsible for the observed high tolerance of this structure to antimicrobials. Surprisingly, the factual body of knowledge concerning in vivo wound biofilm is still rather scant. The majority of data on biofilm properties and tolerance to antimicrobials are derived from ex vivo and in vitro research such as that presented here. The majority of these studies are performed with use of general-purpose microbiological media only. Although the wound fluid immersing factual biofilm differs from these media, it has only been recently that this seemingly obvious fact and phenomena have been related to it, with it now starting to be thoroughly explored [33,34]. Presently, numerous research teams have aimed to develop a method that allows for the wound conditions to be reflected in in vitro conditions as closely as possible [26,35,36,37]. Of note, the media used in biofilm analyses should mimic the composition of wound exudate because host-derived components, which commonly occur in the wound bed, can determine biofilm formation and interact with antimicrobials [33,38]. The wound microenvironment is defined as an extracellular compartment containing cells, growth factors, inflammatory mediators, and host-derived biochemical elements [39]. Depending on the wound healing (or delay of healing) stage, the volume and composition of wound fluid changes. Therefore, also the analysis of the exudate should be conducted in order to accurately mimic the wound environment in vitro [30]. The wound exudate composition is strictly related to serum [32]. The organic (such as proteins, amino acids, glucose) and inorganic components (bicarbonate, calcium, magnesium, chloride, sodium, phosphate) are abundant in the wound exudates [30,31,40]. As a source of many organic and inorganic compounds, fetal bovine serum (FBS) is widely used to supplement DMEM in cell culture. Nevertheless, the FBS composition is not well-defined because of host-dependent specificity. To imitate the wound environment, we used 10% FBS in DMEM in the experiment, corresponding to the medium used for fibroblast culturing. It was already stated that cultures in DMEM with 10% FBS provide optimal cell growth conditions thanks to the nutrients present also in wound exudate [31,32]. The general-purpose laboratory medium—tryptic soy broth (TSB) and tryptic soy broth with 1% glucose (TSB+G)—were used for comparison. The composition of all media applied in the experiment is presented in Table 3. 

In the first line of the experiments, the generally recognized ability of *S. aureus* to form biofilm in vitro was verified (Figures S1–S3), and then the linear correlations between the amount of formed biofilm and metabolic activity of *S. aureus* strains cultured in tested media were assessed (Figure 1 and Figure S4). No linear correlation was found for strains cultured in TSB. However, the positive linear correlation was observed for strains cultured in TSB+G and DMEM, which means that together with the amount of biofilm biomass, the metabolic activity of bacteria also increased. Surprisingly, the correlation coefficient was highest for DMEM. A similar finding was observed for *S. aureus* cultures by Kadam et al. [11], who showed the significant correlation of aforementioned parameters in culture grown in FBS but not in Luria–Bertani broth (LB). In turn, Alonso et al. [41] indicated a poor relationship between total biofilm and cells activity. They confirmed the positive linear correlation for less than 50% of *S. aureus* strains cultured in TSB, while Xu et al. [42] showed the exact correlation for 65% of cases. The results presented in Figure 1 show the shift in direction of higher biomass level in strains cultured in TSB+G, which may be related with increased content of sugar-derived exo-polymeric matrix for which development affects cellular metabolic activity [43], but most importantly they show distinct variations in biofilm formation of the same strains but cultured in various media. Figure 2 presents results of biofilm’s crystal violet staining and metabolic activity measurement in all tested media. These results were compared to see if and how change of the environment affects biofilm formation. There were no visibly and statistically significant differences between the results of the two methods obtained for TSB and TSB+G (Figure 3). This indicates that the specific content of glucose applied in this study did not translate into impact on biofilm formation. Waldrop et al. [44] reported the threshold of glucose concentration above which the biofilm formation increases, indicating that in the case of *S. aureus*, it is 2 g/L. In our research, the glucose concentrations in each medium, according to the manufacturers’ claims, were as follows: TSB—2.5 g/L, TSB+G—10 g/L, DMEM—4.5 g/L. The median glucose concentration in wound fluid was lower than in serum at about 0.2 g/L, probably due to the neutrophil utilization [32]. With regard to results presented by Waldrop et al., we applied higher glucose concentrations than the threshold. However, Lade et al. [45] reported that 0.5% and 1% glucose added to TSB (respectively, 12.5 g/L) strongly promotes *S. aureus* strains to form biofilm. It does not reflect the data obtained in our study and the results of Waldrop; the observed discrepancies could be caused by the strain-specific variability and their genetic differentiation. It should be mentioned that the accessory gene regulator (*agr*), associated with the virulence of *S. aureus,* is regulated by numerous metabolic and environmental factors [46]. The *agr* system is concerned the regulator of biofilm formation and de-attachment. Vuong et al. indicated the impact of *agr* system on the ability of staphylococci to form biofilm on the polystyrene surface [47]. Importantly, it was revealed that glucose deficit activates the *agr* system [48]. However, other studies do not confirm the relationship between *agr* expression and biofilm fomartion [45,49] and postulate the involvement of other gene clusters in discussed process. The glucose can induce bacterial cells to form biofilm by the *gbaAB* operon regulations, and the mutation of *gbaB* results in a decreased glucose-induced biofilm formation [50]. The data on biofilm formation and metabolic activity (Figure 2) in TSB+G showed a higher standard deviation than results obtained for biofilm cultured in TSB and DMEM. Such a discrepancy may be caused by the differences in glucose utilization as an energy substrate by the examined strains. Furthermore, MSSA and MRSA abilities to form biofilm in each medium were compared (Figure 4 and Figure S5). The numerous studies were already carried out to assess the relationship between methicillin resistance occurrence and biofilm formation. One of the most thoroughly investigated mechanisms occurring in *S. aureus* biofilm is the production of polysaccharide intercellular adhesin or polymeric N-acetyl-glucosamine (PIA/PNAG), encoded by *ica* operon. Recently it has been suggested that the mechanism of biofilm formation in MRSA is *ica*-independent and involves protein adhesins. Moreover, it was reported that *ica*-independent biofilm is glucose-induced [51,52]. Our results showed that biofilm formation and metabolic activity is slightly higher for MSSA strains than MRSA. The inverse relationship was observed for cultures grown in DMEM. However, none of these differences was statistically significant, bearing in mind such limitation of the methodology used in our study, as a relatively low number of strains was scrutinized. However, our results correlate with these obtained by other research teams for a greater number of strains. Ghasemian et al. and Tahaei et al. investigated 209 and 300 *S. aureus* strains, respectively [53,54]. Similarly to our results, they found no significant correlation between biofilm formation and methicillin-resistance. Lade et al. also did not obtain significant differences comparing MSSA with MRSA (*n* = 40) and their ability to form biofilm in TSB, as well as in TSB supplemented with glucose [45]. The data presented in Figure 1 and Figure 2 show that medium composition has a significant impact on bacteria growth and biofilm formation [34]. TSB is mainly composed of peptones with a high carbohydrate content, constituting the primary source of carbon and energy. By contrast, DMEM composition is deficient in carbohydrate source, with a predominance of amino acids. When carbohydrate level drops (or it is initially scant), bacteria utilize amino acids, leading to release of ammonia and pH increase [55]. Bacteria are thus forced into active proton acquisition and increase metabolic activity to maintain appropriate value of pH in their cytoplasm. Therefore, nutrient stress induced by medium-specific composition may translate into significant reduction of biofilm formation understood as drop of cell number and matrix level [56]. The increased metabolic activity observed in our study may be the reason for the high correlation between biomass and metabolic activity of biofilms cultured in TSB in comparison to their counterparts cultured in TSB+G (Figure 1). Of note, amino acids are a significant content of the exudate. Depending on the infection factor, the composition of amino acids in exudate differs. In the earlier work of our team, the level and composition of metabolites in exudates collected from patients with non-healing leg ulcers [40] was analyzed. In wound fluids collected from ulcerations colonized/infected by *S. aureus*, the high content of amino acids such as alanine, glutamate, valine, leucine, and isoleucine was found. DMEM contains 16 amino acid compounds, including those detected in our earlier work, i.e. valine, leucine, and isoleucine. Moreover, the studies conducted on the impact of amino acids showed correlation between specific features of biofilm and the amino acid’s conformation. Kolodkin-Gal et al. demonstrated that the D-amino acid mixture containing D-leucine inhibits biofilm formation, whereas the L-amino acid mixture does not affect biofilm [57]. In the presence of L-amino acids, biofilm consisted of robust cell aggregates, while in the presence of D-amino acids, biofilm consisted of thin cellular clusters [58]. Presently, determination of the amino acids’ conformation in wound fluid is a high methodological challenge [40]. Of note, amino acids’ L-forms are commonly found in nature, opposite to D-forms. The impact of amino acids on biofilm formation is significant, and their presence in exudates is established. Therefore, the media applied to investigate biofilm in vitro should contain the specific content of amino acids. From this point of view, general-purpose media such as TSB do not meet this demand. To add another variable to this already complicated equation, it should be noted that L-amino acids reduce ability of yeast-like fungi to form biofilm. It implies, that the effect displayed by amino acids of various conformation on biofilm may vary, depending on the fact as to whether the infective agent is a bacterium, a fungus, or their coculture [59]. 

The inorganic compounds occurring in wound fluids as well in DMEM are bicarbonate, calcium, magnesium, chloride, and phosphate salts [31]. The bicarbonate concentration in wound exudate varies on the phase of wound healing (with value range from 1.47 g/L to 1.6 g/L [31]). The DMEM contains 3.7 g/L of sodium bicarbonate. The present data on impact of bicarbonate on biofilm are derived mainly from research on cystic fibrosis. Dobay et al. reported that the MIC value of bicarbonate for *S. aureus* equals 10.5 g/L, whereas the antibiofilm activity was impossible to determine within the tested range of concentrations [60]. The medium used in the aforementioned report was brain-heart infusion (BHI) supplemented with bicarbonate. In turn, Jaikumpun et al. performed the analyses of a similar type using the artificial sputum medium, with concentration of bicarbonate equal to 2.1 g/L, and showed inhibition of *S. aureus* growth [61]. The compositions of these two media are significantly different, and it satisfactorily explains the discrepancy in results of these two research groups, once again showing the meaning of medium applied on outcome obtained. 

In DMEM, the calcium chloride dihydrate serves as a source of calcium. It was reported that calcium moderates *S. aureus* biofilm structure, leading to decreased thickness [62] and changes in its structure in in vitro conditions [63]. Arrizubieta et al. showed that calcium inhibits staphylococcal biofilm-associated protein (Bap) function by inducing its conformational alteration [64]. Moreover, Abraham et al. proved calcium interaction with a clumping factor B (ClfB) and indicated that a strain’s background is related to the level of observed phenomenon [63]. In our study, we did not determine pH changes occurring in the media in response to the bacterial growth. However, it is a well-established fact that an acidic environment promotes epithelization and fibroblast proliferation. Nevertheless, the infection process increases the pH level to basic value, causing an adverse effect on the tissues, leading to their damage [31].

In the next stage of the study, the efficacy of wound antiseptics on planktonic *S. aureus* cells cultured in different conditions was analyzed (Table 1). In the applied methodology, the highest concentration of antiseptic to possibly obtain was 50% of the initial concentration given by the manufacturer of the antiseptic. The differences in the MIC values obtained for TSB and TSB+G occurred in single strains and were of maximum one dilution factor. The lowest minimal inhibitory concentrations were received for OCT, PHMB, and CHX, regardless of whether it was performed in TSB and TSB+G (Figure 5 and Figure S6). Thus, our results agree with reports indicating high inhibitory effects of these antiseptics, even diluted several times below the working solutions [65]. As mentioned in the first part of the discussion, the supplementary glucose added to the TSB did not correlate with significantly more robust formation of *S. aureus* biofilm. The results also indicate that increased glucose concentrations do not affect the efficacy of applied antiseptics. Of note, MIC values obtained in DMEM differed from these obtained in TSB and TSB+G. As presented in Table 1, MICs of OCT, PHMB, and CHX were significantly lower in DMEM than in TSB and TSB+G. It has been previously reported that the nutrient accessibility in the culture media affects bacteria survival in the presence of antimicrobials [38]. The lower MIC values obtained in DMEM are likely the result of the lower metabolic activity and reduced proliferation of bacterial cells. With regard to MIC, the highest anti-biofilm efficacy was achieved for OCT and CHX (Figure 5, Figures S7 and S8). MBEC values in TSB and TSB+G were comparable, and they were significantly lower than the values obtained for cultures grown in DMEM (Table 2). The high antimicrobial efficacy of CHX has resulted in its widespread use. However, the use of CHX is associated with a risk of side effects, such as allergies or anaphylactic shocks [66]. Moreover, the high cytotoxic effect towards fibroblasts, being the result of exposure to CHX, was also reported [67,68]. The Food and Drug Administration (FDA) released a warning concerning severe allergic reactions caused by CHX applied for wound treatment [66]. The high-grade efficacy for bacterial growth inhibition of PHMB did not translate into analogical effect in terms of biofilm eradication. PHMB eradicated biofilm only in two (TSB) and four times (TSB+G) dilutions of working solution (Figures S7 and S8). The significantly lower concentration of this antiseptic was required to eradicate biofilm formed in DMEM. Interestingly, an inverse relationship between applied media and MIC values was observed for bacteria exposed to PVP-I (Table 1, Figure 5). Significantly, MIC values were higher in DMEM than in other tested media. Assumably, this phenomenon is due to the different mechanism of action of povidone-iodine in comparison with the cases of CHX, OCT, and PHMB. The antimicrobial effect of PVP-I relies on the binding of the iodine molecule to cellular elements [21]. It was revealed that compounds present in the wound bed may also display the affinity to iodine and to reduce the effectiveness of this antiseptic [69]. In the context of in vitro studies, the components of the culture medium may thus be considered potential iodine-binding sites. It could be hypothesized that amino acids, of which a relatively high content is present in DMEM, can bind to iodine, reducing the availability of iodine and nutrients in the cell suspension. Contrary to MIC, MBEC values obtained in DMEM were lower than in TSB and TSB+G among almost half of the tested strains (Figure 5). The difference was one dilution and concerned methicillin-resistant strains. It is important to consider planktonic suspension, which in the applied in vitro setting will eventually form biofilm during 24 h lasting incubation. After this time, the antimicrobial solution is added. Contrary, in case of MIC assessment, the planktonic suspension and antimicrobials are mixed together at the same time-point. During the aforementioned overnight incubation, the biofilm forms to the level restricted by availability of nutrients. Results presented in Figure 2 of this work show that biofilm formed in the DMEM was scant (in the meaning of biomass level compared to biomass level measured for biofilm formed in TSB and TSB+G). Therefore, it may be hypothesized that bacteria are less resistant to treatment with antimicrobials. Similar results were observed by Radischat et al. [70], who compared the efficacy of antiseptics against *Staphylococcus aureus* cultured in ex vivo wound exudate. OCT, PHMB, and PVP-I were applied in the same concentrations as these used in our work. The highest biocidal efficacy was exhibited for OCT, followed by PVP-I, even in settings containing elevated protein content. The antiseptics containing hypochlorites showed the lowest efficacy among tested antimicrobials. No MIC values were determined for GENTA and MICR in the applied range of concentration, including the highest one (50% of the product, respectively, 40 ppm and 20 ppm of total hypochlorite content). The very low content of HOCl/NaOCl in the tested products showcased no microbial growth inhibition in all tested media. As previously reported, chlorine shows antimicrobial efficacy at much higher concentrations, above 670 ppm [27]. Subsequently, we scrutinized the antibiofilm activity of hypochlorite-containing antiseptics. Similarly to the results obtained for planktonic cultures, also in the case of biofilm-forming cells, the application of GRAN and MICR did not lead to eradication of biofilm. It turns out that the antibiofilm effect of GRAN was obtained only for undiluted antiseptics in TSB+G and DMEM (Figure 5, Figures S7 and S8). No MBEC value was determined for strains cultured in TSB. Analogically, even 100% concentration of MICR did not display biocidal effect towards biofilm formed in all tested media. These results stayed in line with the previous reports, which showed that a 50% concentration of MICR displayed no antibiofilm effect [18]. Furthermore, these results are in line with recent reports of Severing et al. [27] and Rembe et al. [26], in which no antibiofilm effect of HOCl/NaOCl was shown. Of note, the incubation of the biofilm at 100% concentration of antiseptic precluded the addition of culturing medium. At that time, the bacteria were deprived of access to nutrients, which affected their metabolic activity and ability to multiply. Therefore, the results of in vitro setting in which 100% of working solution (undiluted product) was applied should be treated with precautions, as their extrapolation on the clinical setting may be valid only in the case of dry wounds devoid of exudate. In all other cases, the presence of exudate leads to the dilution of antiseptic’s initial concentration. 

Gentamycin is the only antibiotic that was analyzed in the experimental setting. The local application of antibiotics is not recommended for wound healing, mostly because of the increasing problem of microbial resistance. Hence one of the acceptable forms of antibiotic used in wound infection is a gentamicin-collagen sponge. In our study, all strains were susceptible to GENTA, according to the antibiogram result (Table S1). GENTA concentration used for a MIC determination corresponded to a garamycin sponge concentration (2 g/L. The MIC values were determined in each medium in the range of concentrations starting from 1 g/L (50% working solution). However, there was no clear trend towards increased or decreased MIC in DMEM compared to TSB or TSB+G (Figure S6). Moreover, no biofilm eradication in the range of gentamicin concentrations tested was found, contrary to previously published work [29,71]. The application of undiluted working solution of gentamycin led to eradication of biofilm of only a few strains, most of which were cultured in DMEM (Figure S7). The mean MIC for GENTA correlates with previously reported values, which varied between tested strains to up to 32 times [71,72]. Our results indicate that the gentamycin efficacy does not depend on the culture media but the strain’s intrinsic sensitivity. The comparison of the MIC (Figure 5 and Figure S6) and MBEC (Figure 5 and Figure S7) values of all tested products showed that the minimal inhibitory concentrations were lower than the biofilm eradication values, staying in line with generally accepted paradigm of biofilm’s high tolerance to antimicrobials [5].

Having obtained MIC and MBEC values, we performed modified antibiofilm dressing’s activity measurement (A.D.A.M.), a more complex method, to mimic the wound environment. The A.D.A.M. method incorporates the release of a compound from bacterial cellulose carrier, which is used as a wound dressing material [73]. The cellulose impregnated with the antimicrobial agent is applied indirectly to the biofilm (reducing risk of non-specific removal of biofilm in result of contact between microbes and carrier surface). Additionally, the aforementioned lack of direct contact imitates to a certain extent the situation in which an antimicrobial compound, after release from the carrier, needs to penetrate and to adhere to the biofilm-forming cells. The method was developed by our team and has been continuously used and developed [35,74,75]. In this study, results for the modified A.D.A.M. method performed in three different culturing media were presented for the first time. Of note, the biofilm formed in DMEM displayed lower cohesiveness to agar disk (applied as growth surface) than biofilms formed in TSB and TSB+G. As a result, the biofilm formed in DMEM was easily rinsed off from the agar surface during the performance of the technique, to the extent to which it did not allow us to record the results, indicating metabolic activity of the bacteria. It indicates notable differences in the amount and quality of biofilm formation depending on the culture medium. The observation forced us to apply a correction to technique principles and to obtain comparable results for antimicrobials tested in DMEM (Figure 6). In each tested media, OCT, PVP-I, and CHX were the most effective compared to the other antimicrobial agents used. Biofilm of specific strains was not eradicated by PHMB, HOCl/NaOCl, and GENTA but showed increased metabolic activity in their presence. For these agents, high standard deviations of the results obtained in the case of specific strains were observed (Figure 6). These intraspecies deviations showed the necessity to include, in the next experimental line, a high number of tested strains to obtain cohesive results. GRAN and MICR displayed lower efficacy towards biofilms formed in DMEM than PHMB and GENTA, which did not correlate with results obtained in TSB and TSB+G. A lack of GRAN and MICR antibiofilm efficacy was confirmed also in previously reported research [18,65]. Surprisingly, MBEC values of GENTA against biofilm formed in DMEM were not obtained; moreover, application of this antibiotic led to higher biofilm eradication than application of PHMB and HOCl/NaOCl. 

In vitro models should be repeatable, cost-effective, and quick to perform. Besides the many limitations already mentioned in this work and by other researchers [76], in vitro biofilm models are necessary for initial screening of antimicrobial compounds of confirmed or alleged activity. The development of adequate in vitro biofilm models represents a significant challenge for researchers. From the one side, the advances of technology allow for more and more accurate reflection of complex conditions in chronic wounds; from the other side, too complex and too sophisticated models make their application restricted to modern, well-equipped laboratories of academia or research and development of corporate departments. The perfect model of wound biofilm should be thus established at the equilibrium point between methodological over-simplicity and over-complexity [77]. In the present work, the relatively simple change of applied culture medium translated into the formation of biofilm of the differentiated level of biomass and metabolic activity was shown. Moreover, the application of specific medium translates into specific outcomes of antimicrobial activity of antiseptics and antibiotic applied for wound treatment. Bearing in mind that results of in vitro studies are frequently used to back up the application of specific antimicrobial in hospital settings, we are convinced that by addressing issues raised in this work, we may sensitize the scientific viewpoint, including the clinical and environmental, on such pivotal matters. 

## 4. Materials and Methods

### 4.1. Microorganisms and Culture Conditions 

For experimental purposes, two reference strains from the American Type Culture Collection (ATCC), *Staphylococcus aureus* 6538 and 33591, and 10 clinical strains isolated from chronic wound infections were chosen. All staphylococcal strains are part of collection of the Department of Pharmaceutical Microbiology and Parasitology of Wroclaw Medical University. Six (five clinical and one reference, 6538) strains were methicillin-susceptible *Staphylococcus aureus* (MSSA) strains, while another six (five clinical and one reference, 33591) were methicillin-resistant *Staphylococcus aureus* (MRSA). 

All experiments were performed in three different media:

- Tryptic soy broth (Biomaxima, Lublin, Poland), later referred to as TSB;
- Tryptic soy broth (Biomaxima, Lublin, Poland) supplemented with 1% glucose (*w*/*v*; Chempur, Piekary Slaskie, Poland), later referred to as TSB+G;
- Dulbecco’s modified Eagle’s medium high glucose (Biowest, Riverside, MO, USA; cat no. L0103) supplemented with 10% of fetal bovine serum (Biowest, Riverside, MO, USA), later referred to as DMEM.

### 4.2. Antimicrobials Applied

The following six antiseptics and one antibiotic were used for experimental purposes:

- Octenisept^®^ (Schülke Mayr GmbH, Vienna, Austria), composed of 0.1% octenidine dihydrochloride, 2% phenoxyethanol, (3-amidpropyl cocoate) dimethylammonium acetate, sodium D gluconate, glycerol 85%, sodium chloride, sodium hydroxide, and purified water, later referred to as OCT;
- Braunol^®^ (B. Braun, Melsungen, Hessen, Germany), composed 7.5% povidone-iodine with 10% available iodine, sodium dihydrogen phosphate dihydrate, sodium iodate, macrogol lauryl ether, sodium hydroxide, and purified water, later referred to as PVP-I;
- Prontosan^®^ wound irrigation solution (B. Braun, Melsungen, Hessen, Germany), composed of purified water, 0.1% betaine surfactant, and 0.1% polyaminopropyl biguanide (polyhexanide), later referred to as PHMB;
- Chlorhexidine digluconate—pharmaceutical raw material (Fagron Pharma Cosmetics, Rotterdam, The Netherlands) to prepare a hydrous solution of final concentration 0.5%, later referred to as CHX;
- Granudacyn^®^ Wound Irrigation Solution (Molnlycke Health Care AB, Göteborg, Sweden), composed of water, sodium chloride, 0.005% sodium hypochlorite, and 0.005% hypochlorous acid, later referred to as GRAN;
- Microdacyn60^®^ Wound Care (Sonoma Pharmaceuticals, Inc, Petaluma, CA, USA), composed of super-oxidized water, sodium chloride, 0.004% sodium hypochlorite, and 0.004% hypochlorous acid, later referred to as MICR;
- Gentamicin sulfate powder (Pol-Aura, Dywity, Poland) to prepare a hydrous solution of final concentration of 0.2% later, later referred to as GENTA.

### 4.3. Assessment of Biofilm Biomass Level Performed Using Crystal Violet Dye (CV) in 96-Well Microtiter Plate

The 0.5 McFarland (MF) density (established using densitometer Densitomat II, BioMerieux, Warsaw, Poland) of the bacteria suspension in specific medium (TSB, TSB+G, DMEM) was prepared and next diluted to 1 × 10^5^ CFU/mL. A total of 100 μL of the suspension was added to the six wells of a 96-well microtiter plate (VWR, Radnor, PA, USA) and incubated for 24 h at 37 °C. Subsequently, the non-adhered cells were removed, and the plate was dried for 10 min at 37 °C. Next, 100 μL of 20% (*v*/*v*) water solution of crystal violet (Aqua-med, Lodz, Poland) was added, and the mixture was incubated for 10 min at room temperature. After incubation, the solution was removed, the biofilm was gently washed twice with 100 μL of 0.9% NaCl (Stanlab, Lublin, Poland), and dried for the next 10 min. A total of 100 μL of 30% water solution of acetic acid (*v*/*v*) (Chempur, Piekary Slaskie, Poland) was then introduced to the plate; the whole setting was subjected to mechanical shaking at 450 rpm for 30 min (Schuttler MTS-4, IKA, Königswinter, Germany). After shaking, the solution was transferred to a fresh plate, and the absorbance of extracted CV was measured at 550 nm wavelength using a MultiScan Go Spectrophotometer (Thermo Fischer Scientific, Waltham, MA, USA). The level of biofilm biomass was performed for each strain in six replications in three independent experiments.

### 4.4. Assessment of Level of Biofilm Metabolic Activity Performed Using Richard’s Method (RM) in a 96-Well Microtiter Plate

The procedures of biofilm inoculation were performed as described in Section 4.3. After removing non-adherent cells, we added 100 μL of tested medium supplemented with 0.1% tetrazolium chloride solution (2,3,5-triphenyl-2H-tetrazolium chloride, TTC) (PanReac AppliChem, Darmstadt, Germany) to each well of the 96-well plate (VWR, Radnor, PA, USA). During the incubation, lasting 2 h at 37 °C, metabolically active cells transformed colorless TTC into red formazan. To dissolve red formazan crystals, we added 100 μL of methanol (Chempur, Piekary Slaskie, Poland), and the plate was shaken at 450 rpm for 30 min (Schuttler MTS-4, IKA, Königswinter, Germany). The solution was transferred to a fresh plate, and the absorbance was measured at 490 nm wavelength (MultiScan Go Spectrophotometer, Thermo Fischer Scientific, Waltham, MA, USA). Six replications for each strain were performed in three independent experiments.

### 4.5. Assessment of the Number of Colony-Forming Units Forming Biofilm Using Quantitative Culture and Richard’s Method

The bacterial suspensions at a density of 0.5 McFarland (MF) (densitometer Densitomat II, BioMerieux, Warsaw, Poland) of two staphylococcal strains (ATCC 6538 and ATCC 33591) in TSB and DMEM were diluted to 1 × 10^5^ CFU/mL and 1 × 10^3^ CFU/mL. Next, 100 µL of each suspension was added to the six wells of two 96-well microtiter plates (VWR, Radnor, PA, USA) and incubated for 24 h at 37 °C. Next, non-adherent cells were gently removed. The biofilm formed on the first plate was dedicated to Richard’s method, as described in Section 4.3. The biofilm on the second plate was dedicated to quantitative culture. To detach biofilm from the wells’ surface, we added 100 µL of freshly prepared 0.1% saponin (VWR Chemicals, Radnor, PA, USA) and resuspended the mixture for 30 s. Next, the content of each well was transferred to 900 µL of 0.9% NaCl (Chempur, Piekary Slaskie, Poland) and vortexed for another 30 s. Then, the serial dilution in saline was performed. A total of 100 µL of selected dilutions were seeded on the tryptic soy agar (Biomaxima, Lublin, Poland) using a glass T-shaped cell spreader (VWR, Radnor, PA, USA). Petri dishes were incubated for 24 h at 37 °C. The colonies were counted visually. 

### 4.6. The Confirmation of Biofilm Formation by Means of Epifluorescent/Confocal Microscopy

The biofilm was inoculated as described in Section 4.3. The medium from biofilm-containing wells of culture plates were removed and replaced with 200 µL of Filmtracer™ LIVE/DEAD™ Bio-film Viability Kit (Invitrogen, Thermo Fisher Scientific, OR, USA) solution and incubated at room temperature for 15 min. After incubation, the solution was removed, and the wells were gently rinsed 3 times with sterile water. Next, the water was removed. The biofilms were analyzed using a confocal microscope Leica SP8 (Leica Microsystems, Wetzlar, Germany) with a 25× water dipping objective using 488 nm laser line and 500–530 nm emission to visualize SYTO-9 and 552 nm laser line and 575–627 nm emission to visualize propidium iodide (PI) in a sequential mode. Images are maximum intensity projections obtained from confocal Z stacks with ≈2 µm spacing in Z dimension. PI is represented in red/orange color, SYTO-9 in green color. The obtained biofilm images were further analyzed using Imaris 9 (Abingdon, UK) software.

### 4.7. Assessment of Antimicrobial Susceptibility of Analyzed Strains to Gentamicin Sulfate Using Disc Diffusion Method

The 24 h cultures of staphylococci strains on Columbia agar plates (Becton, Dickinson and Company, Heidelberg, Germany) were used for the method purpose. Firstly, the bacterial suspension at a density of 0.5 McFarland (densitometer Densitomat II, BioMerieux, Warsaw, Poland) was prepared in sterile 0.9% NaCl (Chempur, Piekary Slaskie, Poland). Next, using a sterile swab stick, the inoculum was seeded on the on the Mueller–Hinton agar (Biomaxima, Lublin, Poland) by streaking the swab three times over the agar surface. A disc impregnated with 10 µg gentamicin (Becton, Dickinson and Company, Sparks, MD, USA) was placed on the inoculated agar plate and incubated for 18 h at 35 °C. After the time period, the inhibition zones were measured and interpreted with the European Committee on Antimicrobial Susceptibility Testing breaking points table [78].

### 4.8. Assessment of Minimal Inhibitory Concentration (MIC) of Applied Antimicrobials Using Spectrometric Assessment and RM in 96-Well Microtiter Plate

A total of 200 μL of the tested antimicrobial product was added to the first well of a row of a 96-well microtiter plate (VWR, Radnor, PA, USA) and diluted geometrically in 100 μL of specific medium. Next, 100 μL of the bacterial suspension of 1 × 10^5^ CFU/mL (prepared as described in Section 4.3) (densitometer Densitomat II, BioMerieux, Warsaw, Poland) was added to wells containing different concentrations of the antimicrobial substance. This methodology enabled us to test a range of antimicrobial concentrations, wherein the highest applied in this work were the following: OCT 0.5 g/L (with phenoxyethanol 10 g/L), CHX 2.5 g/L, PVP-I 37.5 g/L, GRAN 0.05 g/L, MICR 0.04 g/L, PHMB 0.5 g/L (with betaine 0.5 g/L), and GENTA 1 g/L. The control of bacterial growth was bacterial suspension in medium and without antimicrobial agents; the provided control of sterility was medium without bacteria and without antimicrobial agents. Next, the absorbance was measured at 580 nm wavelength (MultiScan Go Spectrophotometer, Thermo Fischer Scientific, Waltham, MA, USA). The plate was incubated at 37 °C for 24 h with shaking at 400 rpm in a plate shaker (Schuttler MTS-4, IKA, Königswinter, Germany). After the incubation, the absorbance was measured again. The lack of measured difference between absorbance values recorded at time 0 and 24 h indicated that concentration of antiseptic in a specific well could be considered an MIC value. To confirm the obtained MIC values, we added 20 μL of 1% tetrazolium chloride salt (2,3,5-triphenyl-2H-tetrazolium chloride) (PanReac AppliChem, Darmstadt, Germany) to each well, and the plate was incubated for 24 h at 37 °C. The MIC value was evaluated by means of this method as the concentration of the compound in a first colorless well after 2 and 24 h of incubation. Three independent experiments consisting of two replicates each were performed to assay the MIC assay.

### 4.9. Assessment of Minimal Biofilm Eradication Concentration (MBEC) of Applied Antimicrobials Using Spectrometric Assessment and RM in a 96-Well Microtiter Plate

In the first step, 100 μL of the bacteria suspension at a density of 1 × 10^5^ CFU/mL (prepared as described in Section 4.3) (densitometer Densitomat II, BioMerieux, Warsaw, Poland) was introduced into the wells of a 96-well test plate (VWR, Radnor, PA, USA) containing 100 μL of the tested medium and incubated for 24 h at 37 °C. The following day, non-adherent cells were removed. The geometric dilutions of antimicrobials in tested media were prepared analogically for MIC assessment and transferred to the plate’s well containing adhered biofilm. After 24 h of incubation at 37 °C, the medium was carefully removed from wells, and 200 μL medium with 0.1% tetrazolium chloride salt (PanReac AppliChem, Darmstadt, Germany) was added. After 2 h incubation at 37 °C, the antimicrobial concentration in the first colorless well, next to the red one, was taken as the MBEC value, and the plate was incubated for another 22 h. Then, the medium was removed, and 200 μL of methanol (Chempur, Piekary Slaskie, Poland) was poured into the wells. After 30 min of shaking the plate at 400 rpm/min (Schuttler MTS-4, IKA, Königswinter, Germany), 100 μL of the solution was transferred to a new plate, and the absorbance was measured at 490 nm wavelength (MultiScan Go Spectrophotometer, Thermo Fischer Scientific, Waltham, MA, USA). The sterility and growth controls were prepared in the manner described for MIC analysis MBEC assessment was performed in six repeats. 

### 4.10. Modified Antibiofilm Dressing’s Activity Measurement (A.D.A.M.)

#### 4.10.1. Preparation of Biocellulose Dressings

The reference strain of *Komagataeibacter xylinus* (Deutsche Sammlung von Mikroorganismen und Zellkulturen—DSM 46602) was cultivated in the Herstin–Schramm (H-S) [79] medium in a 24-well plate (VWR, Radnor, PA, USA) at 28 °C in stationary conditions. After 7 days, bacterial cellulose (BC) was harvested and purified with 0.1 M NaOH (Chempur, Piekary Slaskie, Poland) at 80 °C for 90 min. Then, BC was washed with distilled water until neutral pH value was reached, autoclaved, and stored at 4 °C until further analysis. Next, the sterile dressing was weighted using an analytical balance (Pioneer PA213CM/1, Ohaus, Switzerland) and dried overnight at 60 °C. The weighing was repeated until the weight stopped dropping to estimate the volume of water in BC. To obtain BC dressing impregnated with the tested antimicrobials (BC-S), we placed BC in a well of a 24-well plate with 1 mL of the substance. The plate was stored for 24 h at 4 °C. The BC soaked in 0.9% NaCl (Chempur, Piekary Slaskie, Poland) served as a control (BC-N). The concentration of immersed substance (C) was calculated with Equation (1):

(1) $$C\;=\;100\%\;\times \;\frac{V_{S}}{V_{\mathrm{BC}}+V_{S}}$$

where V_BC_ is the BC volume, and V_S_ is the volume of the substance in the well. 

#### 4.10.2. Antimicrobial Activity of Saturated Dressings

The A.D.A.M. test was performed according to the protocol devised at our laboratory [35] with the modification described by Krzyżek et al. [74]. In the first step, agar discs (5 mm wide and 5 mm thick) were cut out from the sterile agar plate (ChemLand, Stargard, Poland) using a 5 mm diameter cork-borer. Agar disks were placed into the wells in the 24-well test plate (VWR, Radnor, PA, USA). Bacteria suspension was prepared from 24 h culture in a tested medium. Cell density, equal to 1 MF, was established using a densitometer (Densitomat II, BioMerieux, Warsaw, Poland) and diluted to 1 × 10^3^ CFU/mL using a micro-dilution method. A total of 2 mL of prepared suspension was added to agar discs and incubated for 24 h at 37 °C. The following day, 2 mL of 2% agar (VWR Chemicals, Radnor, PA, USA) was poured into a well in a sterile 24-well test plate. After the agar solidified, holes were made using the 5 mm diameter cork-borer. Subsequently, the discs were transferred to the holes in the agar plate in a manner in which the biofilm formed on the discs was at the top of them and the holes were filled with approximately 150 μL of an appropriate medium that form a convex meniscus. The BC-S impregnated with 1mL of the antiseptic/antibiotic were placed on the top of the wells. For a growth control, BC-N were used. The plate was covered with a lid and incubated for 24 h at 37 °C. After the incubation, the BC-S and BC-N were discarded, and the agar discs were gently transferred to a fresh plate. A total of 2 mL of tested medium supplemented with 0.1% tetrazolium chloride solution (2,3,5-triphenyl-2H-tetrazolium chloride, TTC) (PanReac AppliChem, Darmstadt, Germany) was poured into each well, and the plate was incubated for 4 h at 37 °C. The medium was removed and 1 mL of methanol (Chempur, Piekary Slaskie, Poland) was poured into the wells. The plate was shaken at 400 rpm for 30 min (Schuttler MTS-4, IKA, Königswinter, Germany). Finally, 100 μL of the color solution was transferred to a 96-well titration micro-plate (VWR, Radnor, PA, USA) in 6 replicates, and the absorbance was measured at 490 nm wavelength (MultiScan Go Spectrophotometer, Thermo Fischer Scientific, Waltham, MA, USA). 

The coherence of biofilm grown in DMEM was insufficient to perform the test (due to biofilm removal). Therefore, the two final A.D.A.M. steps were modified for biofilms cultured in DMEM. After removal of BC-S and BC-N, the medium above the agar plate was aspirated carefully. Approximately 150 μL of 0.1% TTC solution in DMEM was poured into the hole. The plate was incubated for 4 h at 37 °C. Subsequently, the solution was aspirated, and the agar plate was replaced with the new 24-well plate. The final step was performed as described in the last paragraph. To dissolve formazan crystals, we added 1 mL of methanol to the well, and the plate was shaken at 400 rpm for 30 min. After this time, 100 μL of the solution was moved to the 96-well plate in 6 replicates, and the absorbance was measured (490 nm).

The test was performed in three replicates for each antimicrobial in three independent experiments. All strains were analyzed in TSB, and on the basis of the obtained results, we chose one of the most and one of the least resistant to each tested antimicrobial, as well as reference strains, in order to perform the method in TSB+G and DMEM. The viability of bacterial cells (Vb) was calculated using the following Equation (2):

(2) $$\mathrm{Vb}\;=\;\;100\%\;\times \;\frac{{\mathrm{OD}}_{\mathrm{BC}-S}}{{\mathrm{OD}}_{\mathrm{BC}-N}}\;$$

where OD_BC-S_ is an absorbance value obtained for a sample treated with BC-S, and OD_BC-N_ is the absorbance values of biofilm treated with BC-N. 

### 4.11. Statistical Analysis

Statistical analysis was performed using GraphPad Prism (Version 8.0.1; GraphPad Software Inc., La Jolla, CA, USA, www.graphpad.com (accessed on 1 September 2021)). To identify outliers, we used the ROUT method with a Q value equal to 1%. Normality distribution and variance homogeneity were assessed with the Shapiro–Wilk and Brown–Forsythe tests. The analysis of linear correlation between bacteria’s ability to form biofilm and their metabolic activity was performed using the Pearson or Spearman correlation, depending on the distribution. Additionally, the linear regression was calculated. To compare antimicrobials’ efficacy, we performed non-parametric ANOVA Kruskal–Wallis test with post hoc Dunn’s analysis. Results with a significance level *p* < 0.05 were considered significant. Graphical abstract was created with Biorender.com (accessed on 1 September 2021).

## 5. Conclusions

- The application of various culture media changed the level of staphylococcal biofilm biomass and metabolic activity;
- The staphylococcal biofilms formed in DMEM displayed lower level of biomass and metabolic activity than biofilms formed in TSB and TSB+G;
- The effectiveness of inhibiting bacterial growth and biofilm formation of treatment agents was found to be dependent on type of grown biofilm and the medium applied;
- Results from in vitro studies should be scrutinized carefully with the stress put on the applied methodology.

## Figures and Tables

**Table 1:** Differences in minimal inhibitory concentrations of octenidine dihydrochloride (OCT), chlorhexidine digluconate (CHX), povidone-iodine (PVP-I), polyhexanide (PHMB), and gentamicin sulfate (GENTA) obtained in three media: tryptic soy broth (TSB), tryptic soy broth supplemented with 1% glucose (TSB+G), and Dulbecco’s modified Eagle’s medium (DMEM). The differences were statistically significant for *p* < 0.05 and referred to as *p* < 0.033 (*), *p* < 0.002 (**), *p* < 0.001 (***); ns refers to difference being statistically insignificant. Arrows indicate that in specific medium average MIC values were higher (arrow up) than in another medium (arrow down) toward staphylococcal strains.

Substance	Comparison of MIC Values in Specific Media						
	TSB vs. TSB+G	TSB vs. DMEM			TSB+G vs. DMEM		
OCT	ns	↑	↓	***	↑	↓	***
PVP-I	ns	↓	↑	**	↓	↑	***
CHX	ns	↑	↓	*	↑	↓	*
PHMB	ns	↑	↓	***	↑	↓	***
GENTA	ns	ns			ns		

**Table 2:** Differences in minimal biofilm eradication concentrations (MBEC) of octenidine dihydrochloride (OCT), chlorhexidine digluconate (CHX), povidone-iodine (PVP-I), and polyhexanide (PHMB) obtained in three media: tryptic soy broth (TSB), tryptic soy broth supplemented with 1% glucose (TSB+G), and Dulbecco’s modified Eagle’s medium (DMEM). The differences were statistically significant for *p* < 0.05 and referred to as *p* < 0.033 (*), *p* < 0.002 (**), *p* < 0.001 (***); ns refers to difference being statistically insignificant. Substances for which MBEC was not obtained, such as GRAN, MICR, and GENTA, are not included in the table.

Substance	Comparison of MBEC Values in Specific Media						
	TSB vs. TSB+G	TSB vs. DMEM			TSB+G vs. DMEM		
OCT	ns	↑	↓	***	↑	↓	**
PVP-I	ns	↑	↓	***	↑	↓	*
CHX	ns	↑	↓	**	↑	↓	**
PHMB	ns	↑	↓	***	↑	↓	**

**Table 3:** (**A**) Composition of tryptic soy broth (and tryptic soy broth supplemented with glucose) according to manufacturer’s specification. 10 *—concentration of glucose monohydrate in tryptic soy broth supplemented with glucose (g/L). (**B**) Composition of DMEM.

**(A) Tryptic Soy Broth (TSB)**	**g/L**
*Proteins*	
Pancreatic digest of casein	17
Peptic digest of soybean	3
*Inorganic salts*	
Dipotassium hydrogen phosphate	2.5
Sodium chloride	5
*Other components*	
Glucose monohydrate	2.5/10 *
**(B) Dulbecco’s Modified Eagle’s Medium (DMEM) High Glucose L0103-500 Liquid**	**g/L**
*Amino acids*	
Glycine	0.03
L-Alanyl-L-glutamine (glutamine stable)	0.862
L-Arginine monohydrochloride	0.084
L-Cystine dihydrochloride	0.0626
L-Histidine monohydrochloride monohydrate	0.042
L-Isoleucine	0.105
L-Leucine	0.105
L-Lysine monohydrochloride	0.146
L-Methionine	0.03
L-Phenylalanine	0.066
L-Serine	0.042
L-Threonine	0.095
L-Tryptophan	0.016
L-Tyrosine disodium salt dihydrate	0.10379
L-Valine	0.094
*Inorganic salts*	
Calcium chloride dihydrate	0.265
Ferric nitrate nonahydrate	0.0001
Magnesium sulfate anhydrous	0.09767
Potassium chloride	0.4
Sodium bicarbonate	3.7
Sodium chloride	6.4
Sodium phosphate monobasic anhydrous	0.109
*Vitamins*	
Choline chloride	0.004
D-Ca pantothenate	0.004
Folic acid	0.004
Myo-inositol	0.0072
Nicotinamide	0.004
Pyridoxal hydrochloride	0.004
Riboflavine	0.0004
Thiamine hydrochloride	0.004
*Other components*	
D-Glucose anhydrous	4.5
Phenol red solution salt	0.0159
Sodium pyruvate	0.11

## Data Availability

The data are collected in repository disc and can be shared after a request sent to correspondence author.

## Supplementary Materials

The following are available online at https://www.mdpi.com/article/10.3390/pathogens10111385/s1, Table S1: Zones of inhibition of *Staphylococcus aureus* strains to gentamicin. Figure S1: Live/dead images of *Staphylococcus aureus* biofilm. Figure S2: Live/dead images of cross-sections of *Staphylococcus aureus* biofilm. Figure S3: 3D Live/dead images of *Staphylococcus aureus* biofilm. Figure S4: Linear regression between CFU and RM results for staphylococcal biofilm. Figure S5: Ability to form biofilm of methicillin-susceptible and methicillin-resistant *Staphylococcus aureus* obtained in crystal violet method and Richard’s method. Figure S6: Minimal inhibitory concentrations MIC of CHX, PHMB, 0.008% NaClO/HClO, and GENTA. Figure S7: Minimal biofilm eradication concentrations of CHX, PHMB, 0.008% NaClO/HClO, and GENTA. Figure S8: Survivability of *S. aureus* strains under the different concentrations of tested antimicrobials expressed in CFU%.
