# Supplementary material for: The High Impact of Staphylococcus aureus Biofilm Culture Medium on In Vitro Outcomes of Antimicrobial Activity of Wound Antiseptics and Antibiotic
Source: Pathogens. 2021 Oct 26;10(11):1385. doi: 10.3390/pathogens10111385 (PMC8626063; doi:10.3390/pathogens10111385)
Supplement: Supplementary file 1 [file pathogens-10-01385-s001.zip › pathogens-1386256-supplementary.pdf]

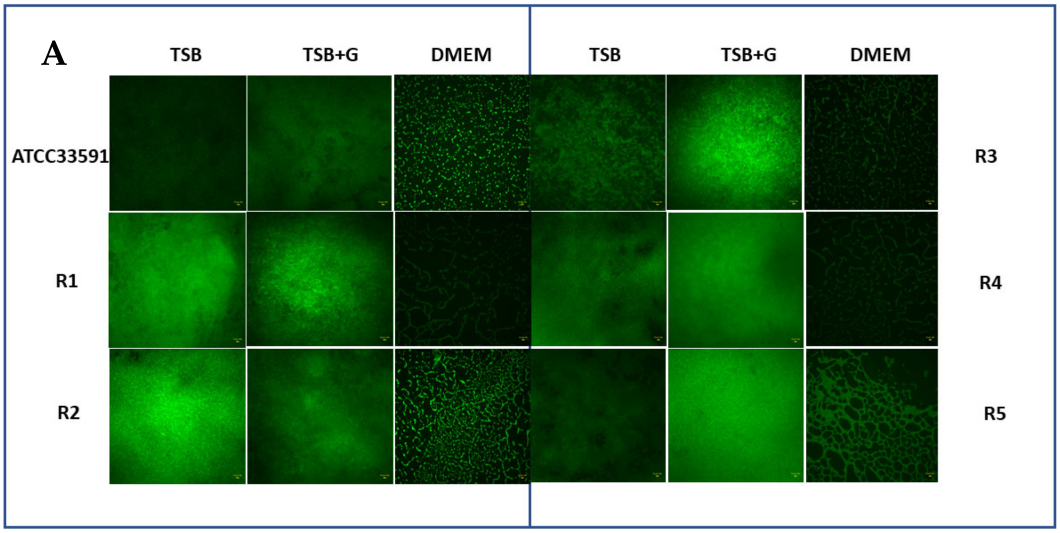

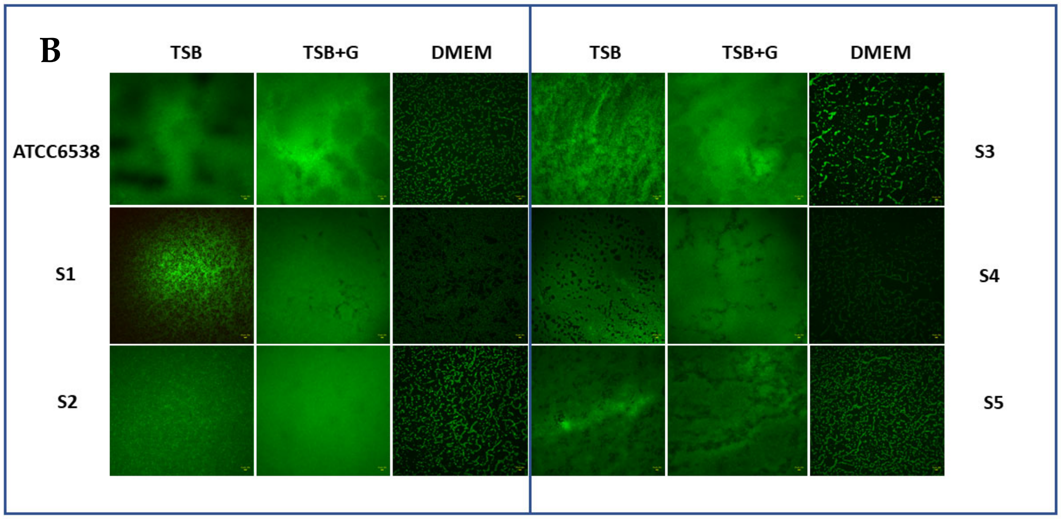

Figure S1. Live / Dead images of *Staphylococcus aureus* biofilm formed in three media: **TSB** - tryptic soy broth, **TSB+G** - tryptic soy broth supplemented with 1% glucose, **DMEM** - Dulbecco's Modified Eagle's Medium; (**A**) methicillin-susceptible (S1-S5 and ATCC 6538) *S. aureus* strains; (**B**) methicillin-resistant (R1-R5 and ATCC 33591) *S. aureus* strains; green—live cells, red/orange—dead cells.

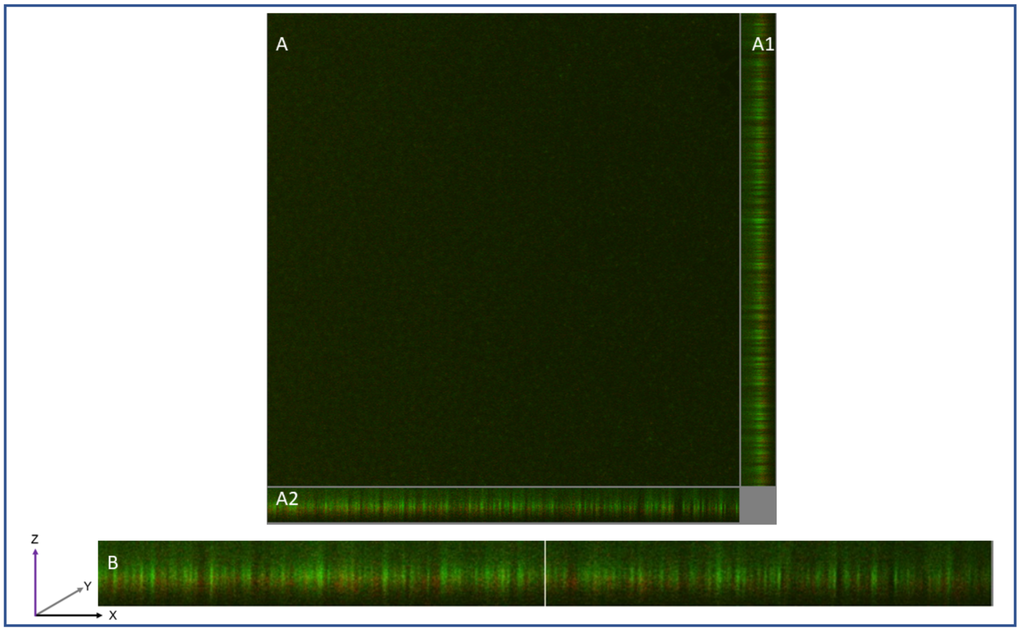

Figure S2 Live / Dead visualization of *Staphylococcus aureus* biofilm (ATCC 33591); (**A**) aerial perspective; (**A1**, **A2**) cross-section of biofilm along Z-axis; (**B**) the Z-axis cross-section enlargement, for the higher visibility of distribution of cell-wall compromised cells (red) and cell-wall intact cells (green).

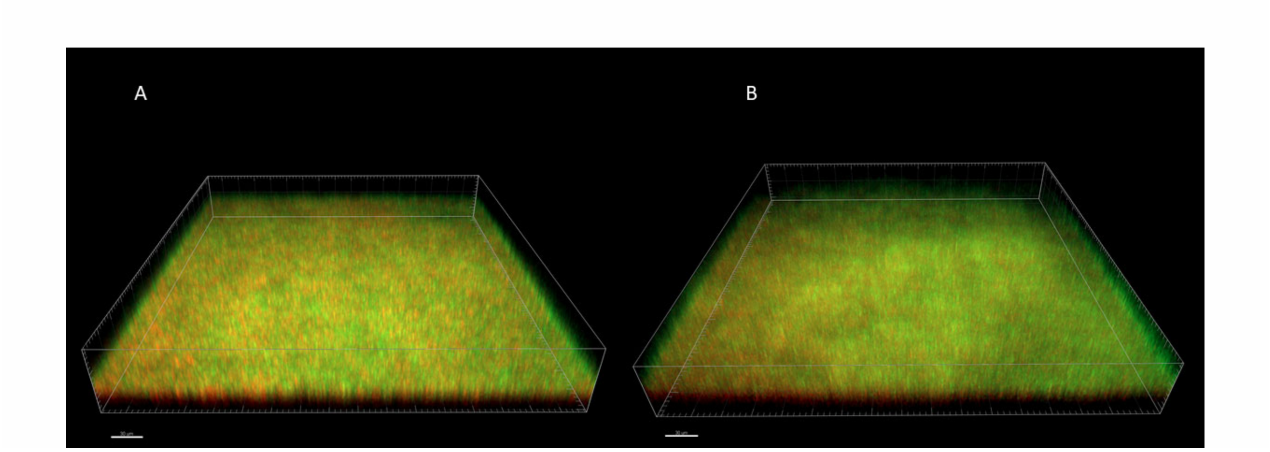

Figure S3 3D Live / Dead visualization of *Staphylococcus aureus* biofilm: (**A**) ATCC6538 and (**B**) ATCC 33591, with the use of Imaris 9.8 software; the side aerial perspective is presented; green—live cells, red/orange—dead cells.

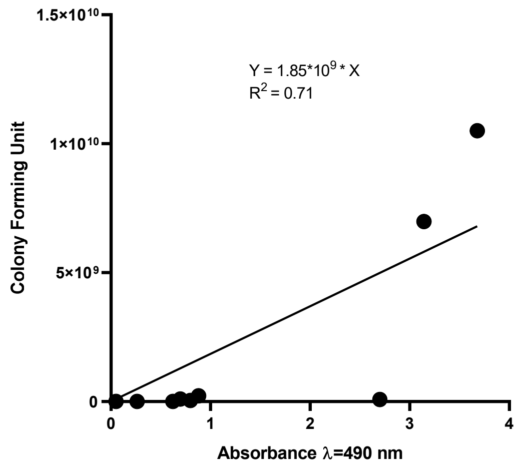

Figure S4 Linear regression between number colony forming units of *Staphylococcus aureus* (axis Y) and its metabolic activity measured in Richard's method and presented as the level of absorbance (axis X); a mathematical equation of linear relationship and coefficient of determination (R^2) are provided.

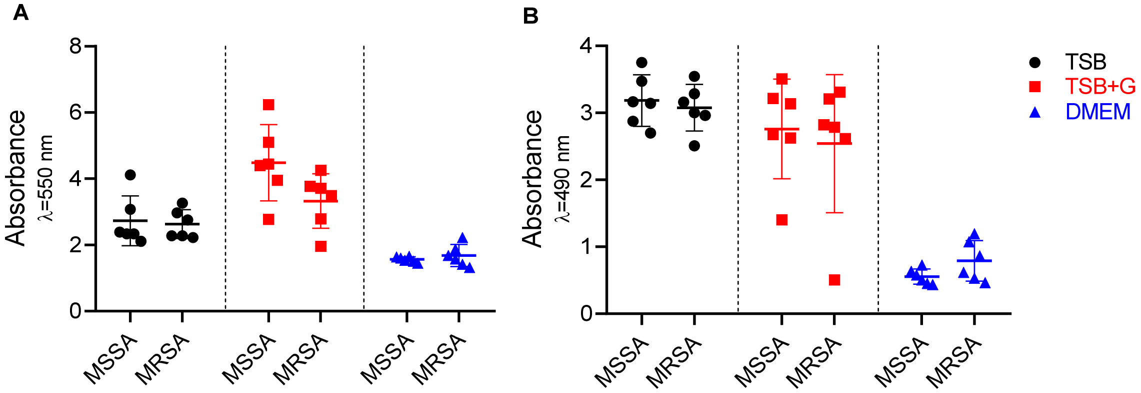

Figure S5 Ability to form biofilm of MSSA - methicillin susceptible *Staphylococcus aureus* and MRSA - Methicillin resistant *Staphylococcus aureus* obtained in two methods: A – Crystal Violet method, B – Richard's method. Strains were cultivated in three media: **TSB** - tryptic soy broth, **TSB+G** - tryptic soy broth with 1% glucose, **DMEM** - Dulbecco's Modified Eagle Medium. An average and standard deviations are marked.

Table S1. Zones of growth inhibition of methicillin-susceptible (S1-S5, ATCC 6538) and methicillin-resistant (R1-R5, ATCC 33591) *Staphylococcus aureus* strains to gentamycin; S – susceptible to gentamicin ATCC – American Type Culture Collection.

Strain	Zone inhibition [mm]	Interpretation*
S1	23	S
S2	22	S
S3	25	S
S4	23	S
S5	25	S
ATCC 6538	26	S
R1	25	S
R2	24	S
R3	26	S
R4	23	S
R5	25	S
ATCC 33591	25	S

* interpreted with the European Committee on Antimicrobial Susceptibility Testing. Breakpoint tables for interpretation of MICs and zone diameters, version 11.0, 2021

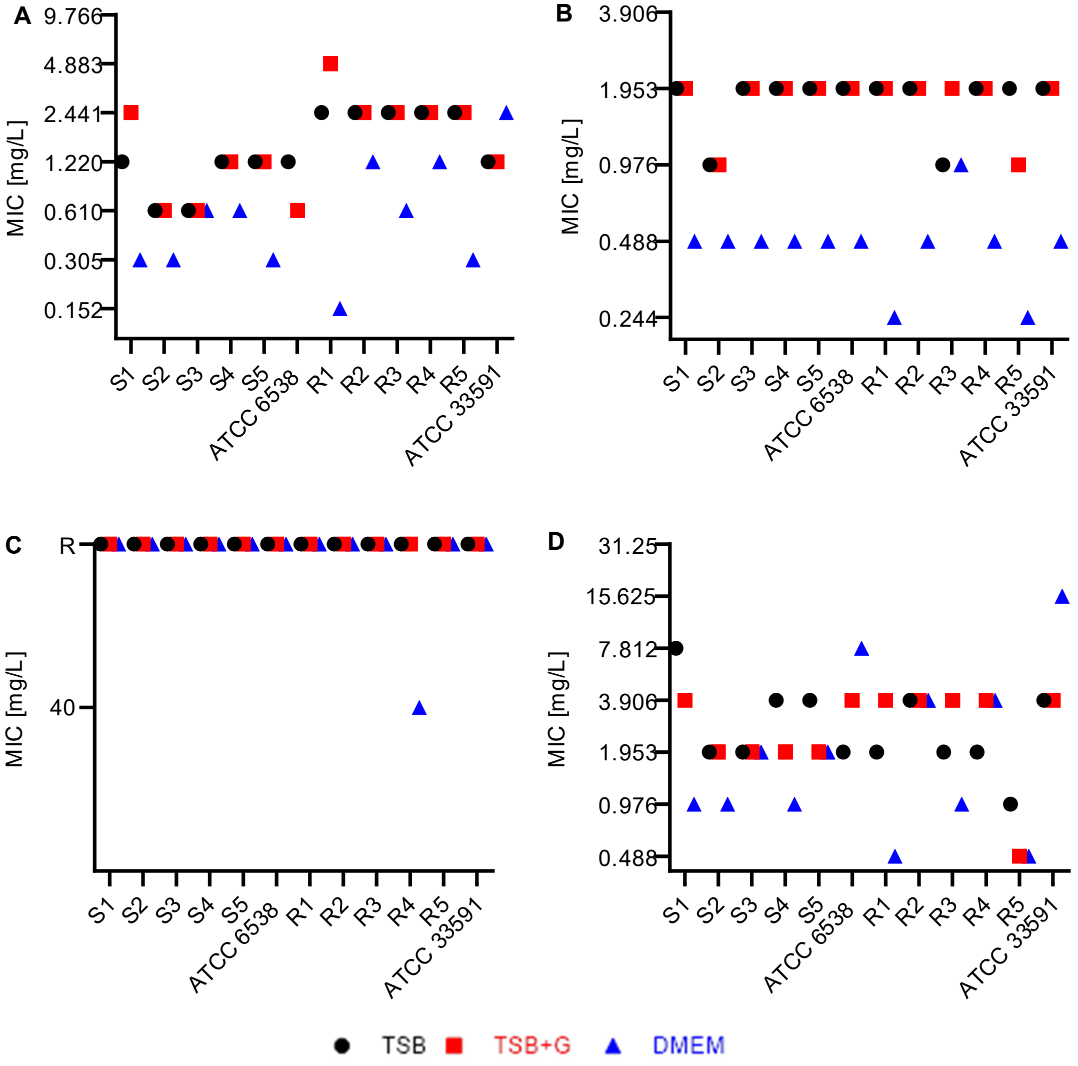

Figure S6 Minimal inhibitory concentrations MIC of **A** – **CHX** chlorhexidine digluconate, **B** - **PHMB** polyhexanide, **C** – **0.008% NaClO/HClO** antimicrobial agent, **D** – **GENTA** gentamycin sulphate of methicillin-susceptible (S1-S5), methicillin-resistance (R1-R2) and type (ATCC American Type Culture Collection) *Staphylococcus aureus* strains. Concentrations are presented in mg/L; **TSB** – tryptic soy broth, **TSB+G** - tryptic soy broth supplemented with 1% glucose, **DMEM** - Dulbecco's Modified Eagle's Medium; R – resistant to the highest tested concentration.

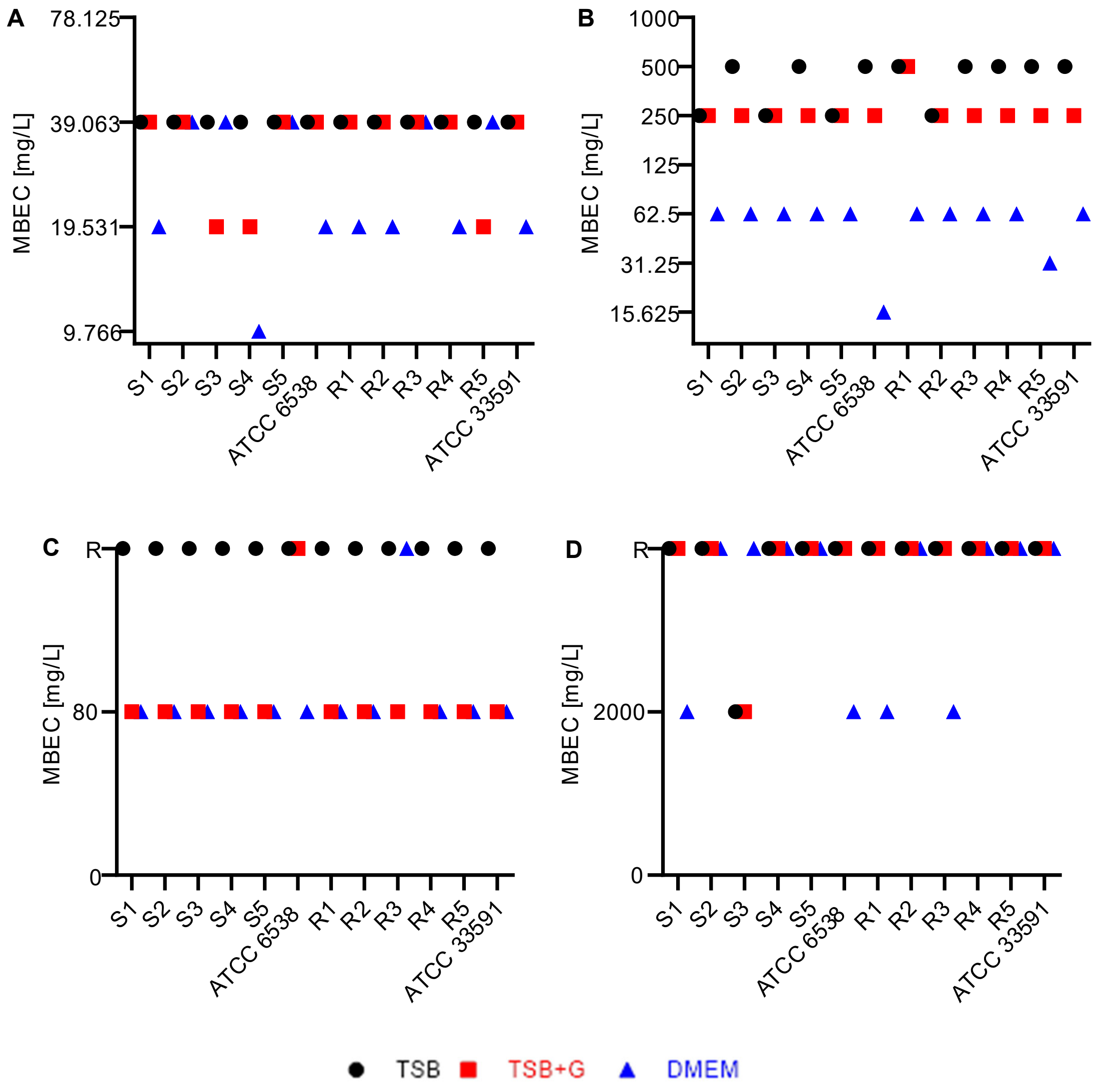

Figure S7. Minimal biofilm eradication concentrations MBEC of **A** – **CHX** chlorhexidine digluconate, **B** - **PHMB** polyhexanide, **C** – **0.008% NaClO/HClO** antimicrobial agent, **D** – **GENTA** gentamycin sulphate of methicillin-susceptible (S1-S5), methicillin-resistance (R1-R2) and type (ATCC American Type Culture Collection) *Staphylococcus aureus* strains. Concentrations are presented in mg/L; **TSB** – tryptic soy broth, **TSB+G** tryptic soy broth supplemented with 1% glucose, **DMEM** - Dulbecco's Modified Eagle's Medium; R – resistant to the highest tested concentration.

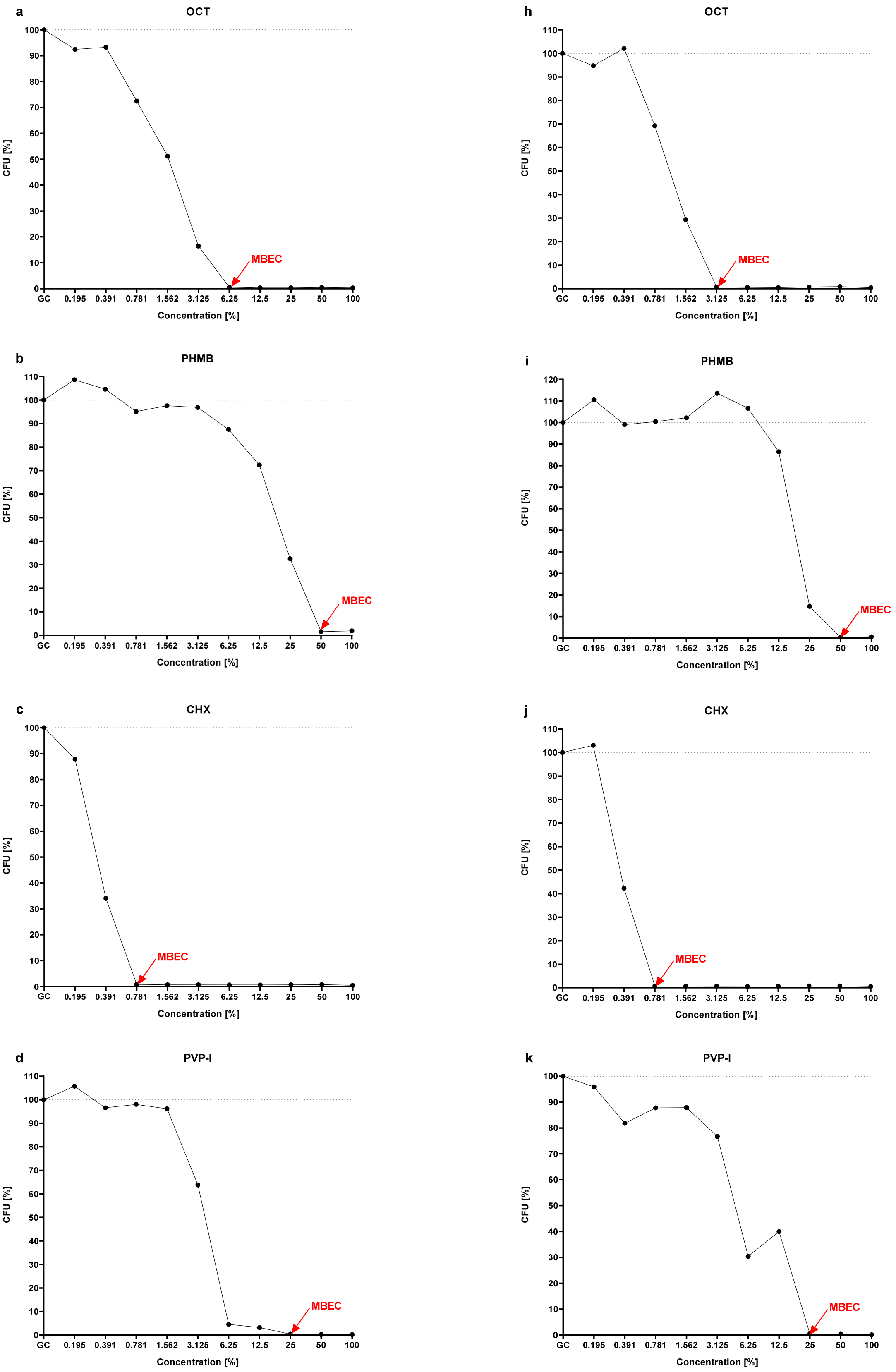
a
OCT
b
PHMB
c
CHX
d
PVP-I
h
OCT
i
PHMB
j
CHX
k
PVP-I
CFU [%]
Concentration [%]
GC
0.195
0.391
0.781
1.562
3.125
6.25
12.5
25
50
100
MBEC

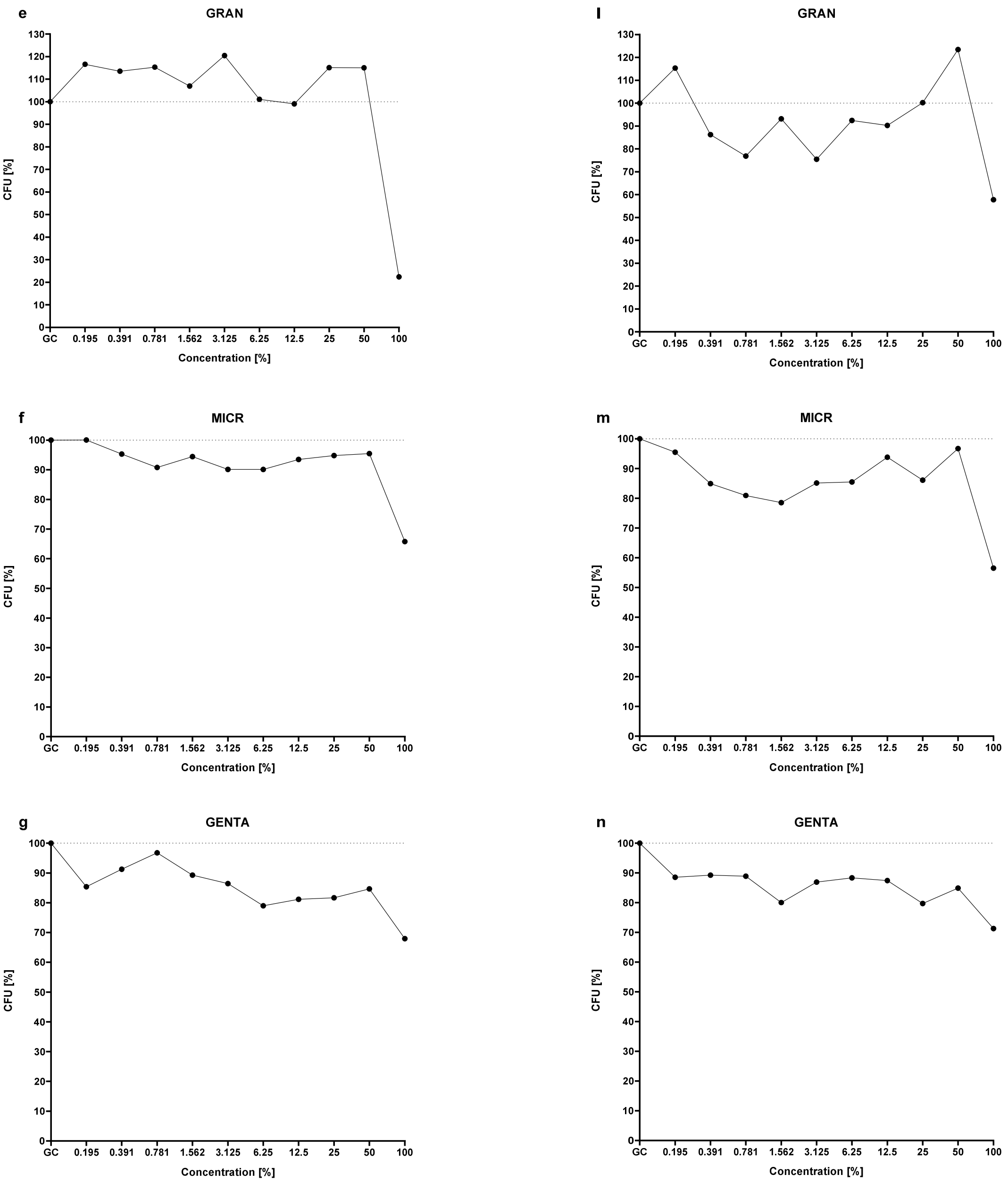

Figure S8 Survivability of methicillin-susceptible ATCC 6538 (a, b, c, d, e, f, g) and methicillin-resistant ATCC 33591 (h, i, j, k, l, m, n) strains of *Staphylococcus aureus* cultured in tryptic soy broth presented as a percentage of Colony Forming Units CFU in comparision to the growth control (**GC**) under a range of dilutions of tested substances; **OCT** octenidine dihydrochloride (a,h), **PHMB** polyhexanide (b, i), **CHX** chlorhexidine digluconate (c, j), **PVP-I** povidone-iodine (d, k), **GRAN** 0,01% NaClO/HClO antimicrobial agent (e, l), **MICR** 0,008% NaClO/HClO antimicrobial agent (f, m), **GENTA** gentamicin sulfate (g, n); ATCC – American Type Culture Collection; red arrows show MBEC values recorded by TTC and quantitative culturing methods.
